# Metabolic Interactions of Side-chain Extended and Unsaturated Vitamin D Analogs with Cytochrome P450 Enzymes: Integrating Theoretical and Experimental Approaches

**DOI:** 10.3390/biom15091222

**Published:** 2025-08-25

**Authors:** Teresa Żołek, Mayur Kadam, Sharmin Nadkarni, Kaori Yasuda, Michał Chodyński, Krzysztof Krajewski, Olga Michalak, Joanna Tobiasz, Marek Kubiszewski, Toshiyuki Sakaki, Andrzej Kutner

**Affiliations:** 1 Department of Organic and Physical Chemistry, Faculty of Pharmacy, Medical University of Warsaw, 1 Banacha, 02-097 Warsaw, Poland; 2 Department of Drug Chemistry, Pharmaceutical and Biomedical Analysis, Faculty of Pharmacy, Medical University of Warsaw, 1 Banacha, 02-097 Warsaw, Poland; mayur.kadam@wum.edu.pl; 3 Department of Chemistry, Sant Rawool Maharaj Mahavidyalaya S. N. Desai Chowk, Udyamnagar, Kudal, Sindhudurg, Maharashtra 416520, India; crl@srmcollege.in; 4 Department of Pharmaceutical Engineering, Toyama Prefectural University, Toyama 939-0398, Japan; kyasuda@pu-toyama.ac.jp (K.Y.); tsakaki@pu-toyama.ac.jp (T.S.); 5 Chemistry Section, Research Group of Pharmacy, Cosmetic Chemistry and Biotechnology, Łukaszewicz Research Network-Industrial Chemistry Institute, 8 Rydygiera, 01-793 Warsaw, Poland; michal.chodynski@ichp.lukasiewicz.gov.pl (M.C.); krzysztof.krajewski@ichp.lukasiewicz.gov.pl (K.K.); olga.michalak@ichp.lukasiewicz.gov.pl (O.M.); joanna.tobiasz@ichp.lukasiewicz.gov.pl (J.T.); marek.kubiszewski@ichp.lukasiewicz.gov.pl (M.K.)

**Keywords:** vitamin D analog, cytochrome CYP3A4, cytochrome CYP24A1, computational modeling, PRI-1906, PRI-1927, PRI-1937, PRI-1938

## Abstract

The clinical use of 1,25-dihydroxycholecalciferol (1,25D3), the active form of vitamin D_3_, is limited by its calcemic side effects and rapid metabolic degradation. To overcome these limitations, we designed novel vitamin D analogs with extended, rigidified, and branched side chains. Among them, **PRI-1938**, featuring a 5,6-*trans* triene system and 22,24-all-*trans* side-chain geometry, demonstrated markedly enhanced resistance to enzymatic catabolism. In vitro assays revealed that metabolic conversion of **PRI-1938** by the nonselective cytochrome P450 3A4 (CYP3A4) enzyme was ca. 4-fold lower than that of the previously obtained **PRI-1906** and over 9-fold lower than 1,25D3. All new analogs, including **PRI-1927** and **PRI-1937**, exhibited significantly higher stability toward mitochondrial cytochrome P450 24A1 (CYP24A1), the vitamin D-selective catabolic enzyme, than that of 1,25D3. Molecular modeling and quantum mechanical calculations indicated that **PRI-1938** adopts a highly stable conformation in the CYP24A1 active site, stabilized by four hydrogen bonds and multiple hydrophobic interactions. The spatially optimized interaction network reduces access to the catalytic heme, resulting in the lowest observed metabolic conversion. These findings highlight the critical role of the side-chain geometry in modulating metabolic stability and support the further development of **PRI-1938** as a promising anticancer vitamin D analog.

## 1. Introduction

**Figure 1:**
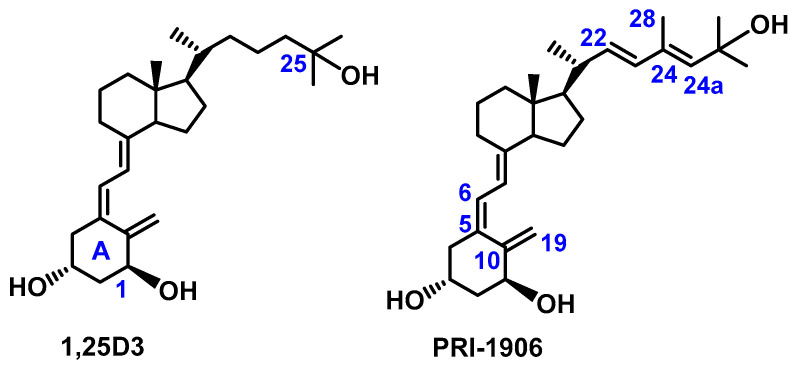
Chemical structure of 1,25-dihydroxyvitamin D_3_ (1,25D3) and its leading analog **PRI-1906**.

1,25-dihydroxycholecalciferol (Figure 1, 1,25D3) [1] is the biologically active form of vitamin D_3_ [2]. 1,25D3 acts as a nuclear hormone, activating the vitamin D receptor (VDR) as a transcription factor to regulate gene expression. Physiologically, 1,25D3 regulates calcium and phosphate homeostasis, maintains bone mineralization, modulates immune responses to infections, and prevents autoimmune diseases [3]. The clinical use of 1,25D3 is limited by its calcemic side effects and rapid metabolic degradation. Consequently, novel vitamin D analogs are being developed to minimize calcemic effects while enhancing metabolic resistance, antiproliferative, pro-differentiating, and immunomodulatory properties compared to 1,25D3. In contrast, metabolically labile analogs of 1,25D3 are intentionally employed in the topical treatment of psoriasis to minimize systemic adverse effects such as hypercalcemia. Analogs of 1,25D3 modified in a single point of their structure typically feature alterations in the A ring or the side chain [4]. Substitutions at C-2 in the A ring [5], modifications in side-chain geometry, and side-chain extension have proven particularly effective. Combining these modifications yielded double-point modified analogs [6,7] with enhanced VDR affinity, showing promise for applications in autoimmune skin disease (e.g., psoriasis), bone disease (e.g., osteoporosis) [8], and anti-hyperproliferative therapies (e.g., cancer) [9]. These analogs exhibit improved metabolic stability and reduced calcemic toxicity.

As part of our research on vitamin D analogs with enhanced tumor cell differentiation and antiproliferative activity, we previously synthesized a 1,25D3 analog, **PRI-1906** (Figure 1), and related compounds [10] with an extended and rigidified side-chain. **PRI-1906** demonstrated over 7-fold greater differentiation activity in human leukemia HL-60 cells compared to 1,25D3. Despite a 25-fold lower VDR binding affinity compared to 1,25D3, **PRI-1906** exhibited high metabolic resistance to CYP24A1 [11]. Mitochondrial cytochrome P450 24A1 (CYP24A1) is the primary enzyme hydroxylating 1,25D3 [2]. C-24 hydroxylation, catalyzed by CYP24A1, initiates vitamin D catabolism, producing progressively fewer active metabolites, ultimately yielding inactive forms such as calcitroic acid [1]. The CYP24A1 gene is the most highly upregulated by ligand-bound VDR [12], with low baseline expression in the absence of 1,25D3 [13].

Although the full-length three-dimensional (3D) structure of human CYP24A1 (*h*CYP24A1) remains unresolved, the rat CYP24A1 crystal structure (*r*CYP24A1: PDB: 3K9V, 2.5 Å resolution) [14] and homology models provide insights into its active site. Key residues, including Ile131, Trp134, Met148, Met246, Phe249, Ala326, Val391, Thr394, Thr395, Gly499, and Ile500, govern substrate binding and C-24/C-23 hydroxylation selectivity for 1,25D3 and analogs like **PRI-1906**. In contrast, the 3D structure of human cytochrome P450 3A4 (*h*CYP3A4; PDB: 2V0M, 2.8 Å resolution) [11] is well-characterized, facilitating studies of its broad metabolic activity. Structurally, *r*CYP24A1 exhibits the canonical cytochrome P450 fold, with 12α-helices (A–L), four β-sheet systems (β1–β4), and additional helices A′, B′, G′, K′, and K″ located on the distal surface and between β2 and the heme-binding motif, respectively [14]. Its F-helix, spanning 18 residues, lacks the distinct F′-helix observed in *h*CYP3A4. Conversely, *h*CYP3A4 features a flexible active site with a well-defined F′–G′ loop and a larger substrate-binding cavity, facilitating metabolism of diverse substrates, including nearly half of all drugs. The compact active site of *r*CYP24A1, shaped by the absence of a structured F′-helix, is optimized for specific vitamin D metabolite interactions, whereas *h*CYP3A4’s open conformation supports its promiscuous substrate specificity. To investigate metabolic interactions, we previously performed rigid docking of vitamin D analogs to *h*CYP3A4, validated by molecular dynamics (MD) simulations, to predict their metabolic conversion [11]. The calculated binding free energies of 1,25-dihydroxyvitamin D_2_ (1,25D2) and 1,25D3 analogs to *h*CYP3A4 correlated with their experimental conversion by *h*CYP24A1, allowing for the prediction of *r*CYP24A1-mediated metabolism despite the unresolved structure of *h*CYP24A1. These findings highlight significant differences in the metabolic profiles of side-chain-modified analogs, underscoring the importance of structural and computational approaches in evaluating novel vitamin D analogs.

**Figure 2:**
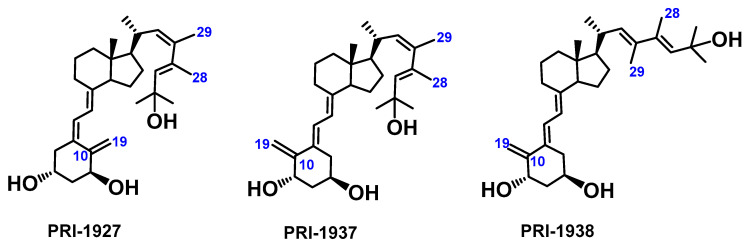
Chemical structure of the new analogs of 1,25D3: **PRI-1927**, **PRI-1937**, and **PRI-1938**.

Recently, 1,25D3 analogs containing a (22*R*)-methyl substituent in the side chain were synthesized [8]. These analogs exhibited high calcemic activity, with strong in vivo effects on bone and elevated intestinal calcium transport. In contrast, our **PRI-1906**, with a C-24 methyl, increased serum calcium level by only half that of 1,25D3, though residual calcemic activity limits its clinical utility. Motivated by **PRI-1906’s** reduced calcemic effects, we further modified the side chain to enhance branching. In this study, we designed and characterized isomeric homologs of **PRI-1906** (**PRI-1927**, **PRI-1937**, and **PRI-1938**) (Figure 2), featuring extensively branched side chains and variations in side chain and triene system geometries to optimize selective interactions with VDR LBP. The metabolic stability of these analogs, alongside **PRI-1906**, was evaluated against *h*CYP3A4 and compared with the *h*CYP24A1 assay. The experimental data were correlated with the theoretical calculations, including rigid docking and MD simulations of *r*CYP24A1 and *h*CYP3A4. In summary, molecular modeling studies have elucidated the structural determinants of vitamin D_3_ analog interactions with *r*CYP24A1 and *h*CYP3A4, highlighting the impact of side chain branching and A-ring modifications on metabolic stability and receptor affinity. These findings, supported by advanced computational methods, provide a foundation for designing novel analogs with tailored therapeutic profiles.

## 2. Materials and Methods

### 2.1. Experimental

#### 2.1.1. General Procedures

Solvents for reactions, flash chromatography, and HPLC, chemicals, and reagents were purchased from commercial sources and used without further purification. Infrared (IR) spectra were recorded on an FT-IR spectrophotometer as films of oily substances. Flash column chromatography (FCC) was conducted on silica gel Si 60 (230–400 mesh, Merck, Darmstadt, Germany).

#### 2.1.2. Mass Spectrometry and NMR Spectra

High-resolution mass spectrometry measurements were performed using the Synapt G2–Si mass spectrometer (Waters), equipped with an electrospray ionization (ESI) source and a quadrupole time-of-flight (Q-TOF) mass analyzer or Q-Exactive hybrid quadrupole-orbitrap mass spectrometer system equipped with a heat electrospray ionization (HESI) source. Measurement results were processed with the MassLynx 4.1 software (Waters). The ^1^H NMR (500 MHz) and ^13^C NMR (125 MHz) spectra were obtained in CD_3_OD solutions on a Bruker AVANCE III HD 500 MHz spectrometer at 298 K. Chemical shifts for ^1^H and ^13^C NMR are given relative to the TMS signal at δ = 0.0 ppm. One-dimensional and two-dimensional NMR experiments were performed for structure analysis.

#### 2.1.3. HPLC Chromatography and UV Spectroscopy

Analytical HPLC separation was carried out using reverse-phase high-performance liquid chromatography (HPLC) on a Purospher^®^ Star RP-18 Endcapped column (4.6 × 250 mm, 5 μm) at 40 °C. The analysis employed a linear acetonitrile–water gradient (20–100% CH_3_CN, 0–25 min) at a flow rate of 1 mL/min, with UV detection at 265 nm. UV spectra were recorded at the apex of each peak. Preparative HPLC separation was performed on a Chromolith^®^ SemiPrep RP-18e column (10 × 100 mm) at 40 °C. A linear acetonitrile–water gradient (50–100% CH_3_CN, 0–20 min) was applied at a flow rate of 4.7 mL/min. The injected sample (20 mg/mL) had a volume of 100–150 μL. The collected fractions were dried under reduced pressure.

### 2.2. Syntheses

#### 2.2.1. (E)-2,4-dimethylhex-3-ene-2,5-diol (**2**)

To a solution of **1** (1.0 g, 7.00 mmol, 1.0 equiv.) in anhydrous THF (5.0 mL) at 0 °C was added MeMgBr (3.0 M in THF, 7.5 mL, 22.5 mmol, 3.2 equiv.) dropwise, and the solution was stirred at 0 °C for 1 h. The resulting mixture was stirred at 25 °C for 3 h, and then quenched with saturated aq. NaHCO_3_ solution (20 mL). The layers were separated, and the aqueous phase was extracted with *tert*-butyl methyl ether (*t*-BuOMe; 3 × 10 mL). The combined organic extracts were washed with brine (20 mL), dried over anhydrous Na_2_SO_4_, filtered, and concentrated under reduced pressure. The crude material was purified by FCC using 15% EtOAc/hexane, affording product **2** as a colorless oil (600 mg, 60%). **^1^H NMR** δ: 5.55 (1H, s, H-3), 4.09 (1H, q, J = 6.5 Hz, H-5), 1.83 (3H, s, H-7), 1.36 (3H, s, H-1 or H-8), 1.35 (3H, s, H-1 or H-8), 1.23 (3H, d, J = 6.5 Hz, H-6). **^13^C NMR** δ: 139.2 (C-4), 131.4 (C-3), 73.2 (C-5), 70.0 (C-2), 29.6 (C-1 or C-8), 29.5 (C-1 or C-8), 20.6 (C-6), 11.0 (C-7). **FT-IR** (cm^−1^): 3277, 2969, 1664, 1365, 1189, 1079. **HRMS** (HESI): calc’d for C_8_H_16_O_2_ [M+H]^+^ 145.1150, found 145.1220.

#### 2.2.2. (E)-5-hydroxy-3,5-dimethylhex-3-en-2-one (**3**)

To a solution of **2** (650 mg, 4.17 mmol, 1.0 equiv.) in DCM (2.0 mL) at 0 °C was added DMP (1.76 g, 4.17 mmol, 1.0 equiv.) lot-wise, and the solution was stirred at 0 °C for 10 min. The resulting mixture was stirred at 25 °C for 2 h. The reaction mixture was quenched with sat. aq. NH_4_Cl solution (10 mL) and the layers were separated. The aqueous phase was extracted with DCM (3 × 10 mL), and the combined organic extract was washed with brine (10 mL), dried over anhydrous Na_2_SO_4_, filtered, and concentrated under reduced pressure. The crude product was purified by FCC using 8% EtOAc/hexane, affording product **3** as a colorless oil (450 mg, 70%). **^1^H NMR** δ [ppm]: 6.80 (1H, m, H-4), 2.32 (3H, s, H-1), 1.97 (3H, d, J = 1.3 Hz, H-8), 1.44 (6H, s, H-6 and H-7). **^13^C NMR** δ [ppm]: 203.3 (C-2), 151.1 (C-4), 137.9 (C-3), 71.7 (C-5), 30.0 (C-6 and C-7), 25.8 (C-1), 11.8 (C-8). **FT-IR** (cm^−1^): 3432, 2974, 1663, 1366, 1173, 1025. **HRMS** (HESI): calc’d for C_8_H_14_O_2_ [M+H]^+^ 142.0994, found 143.1067.

#### 2.2.3. (E)-3,5-dimethyl-5-((triethylsilyl)oxy)hex-3-en-2-one (**4**)

To a solution of 3 (400 mg, 2.81 mmol, 1.0 equiv.) in DCM (2.0 mL) at 0 °C was added imidazole (Im, 479 mg, 7.04 mmol, 2.5 equiv.) and triethylsilyl chloride (TESCl) (0.71 mL, 4.22 mmol, 1.5 equiv.). The mixture was stirred at 0 °C for 10 min, and then the resulting mixture was stirred at 25 °C for 2 h. The reaction mixture was quenched with sat. aq. NH_4_Cl solution (10 mL) and the layers were separated. The aqueous phase was extracted with DCM (3 × 8 mL), and the combined organic extract was washed with brine (10 mL), dried over Na_2_SO_4_, filtered, and concentrated under reduced pressure. The crude product was purified by FCC using 1% EtOAc/hexane, affording ketone **4** as a colorless oil (450 mg, 62%). **^1^H NMR** δ [ppm]: 6.78 (1H, d, J = 1.3 Hz, H-4), 2.32 (3H, s, H-1), 1.96 (3H, d, J = 1.3 Hz, H-8), 1.49 (6H, s, H-6 and H-7), 1.00 (9H, t, J = 8.0 Hz, H-11), 0.66 (6H, k, J = 8.0 Hz, H-10). **^13^C NMR** δ [ppm]: 203.4 (C-2), 151.7 (C-4), 137.9 (C-3), 74.7 (C-5), 31.3 (C-6 and C-7), 25.8 (C-1), 12.1 (C-8), 7.6 (C-10), 7.4 (C-11). **FT-IR** (cm^−1^): 2956, 1674, 1229, 1168, 1092, 1042. **HRMS** (ESI): calc’d for C_14_H_28_O_2_Si [M+H]^+^ 256.1859, found 256.1862.

#### 2.2.4. (1S,3R,5E,7E,22Z,24E)-24-Dehydro-24a-homo-23-methyl-1,3-bis-(t-butyldimethylsilyl)-25-(triethylsilyl)ergocalciferol (**6**) and (1S,3R,5E,7E,22E,24E)-24-dehydro-24a-homo-23-methyl-1,3-bis-(t-butyldimethylsilyl)-25-(triethylsilyl)ergocalciferol (**7**)

To a solution of **5** (120 mg, 0.159 mmol, 1.0 equiv.) in anhydrous THF (1.0 mL) at −78 °C was added lithium hexamethyldisilazide (LiHMDS, 1.0 M in THF, 180 μL, 0.180 mmol, 1.1 equiv.) dropwise. The reaction mixture was stirred at −78 °C for 30 min. Then **4** (50 mg, 0.190 mmol, 1.2 equiv.) in anhydrous THF (0.3 mL) was added dropwise. The reaction mixture was stirred at −78 °C for 30 min, and then at 25 °C for 4 h. The reaction mixture was quenched with sat. aq. NH_4_Cl solution (5 mL), and the layers were separated. The aqueous phase was extracted with *t*-BuOMe (3 × 10 mL). The organic extract was washed with brine (10 mL), dried over anhydrous Na_2_SO_4_, filtered, and concentrated under reduced pressure. The crude product was purified by FCC using hexane, affording a mixture of **6** and **7** as a colorless oil (55 mg, 52%). The mixture was used for the next step without separation.

#### 2.2.5. (1S,3R,5E,7E,22Z,24E)-24-Dehydro-24a-homo-23-methyl-1,25-dihydroxyergocalciferol (**PRI-1937**) and (1S,3R,5E,7E,22E,24E)-24-dehydro-24a-homo-23-methyl-1,25-dihydroxyergocalciferol (**PRI-1938**)

To a solution of **6** and **7** (25 mg, 0.031 mmol, 1.0 equiv.) in anhydrous THF (0.3 mL) at 50 °C was added TBAF (1.0 M in THF, 32 μL, 0.032 mmol, 1.02 equiv.). The mixture was stirred at 50 °C for 2 h, and the solvent was removed under reduced pressure. The crude product was purified by FCC (using 35% EtOAc/hexane), affording a mixture of **PRI-1937** and **PRI-1938**. The analogs were separated by preparative HPLC, affording **PRI-1937** as a colorless oil (7.6 mg, 53.2%) and **PRI-1938** as a colorless oil (0.8 mg, 5.6%). **PRI-1937**: **^1^H NMR** δ [ppm]: 6.54 (1H, d, J = 11.5 Hz, H-6), 5.92 (1H, d, J = 11.5 Hz, H-7), 5.29 (1H, d, J = 1.2 Hz, H-24a), 5.06 (1H, d, J = 1.7 Hz, H-19), 4.96 (1H, s, H-19), 4.90 (1H, dd (overlaps with OH of solvent), H-22), 4.44 (1H, m, H-1), 4.14 (1H, m, H-3), 2.90 (1H, m, H-9), 2.73 (1H, m, H-4), 2.50 (1H, m, H-20), 2.28 (1H, m, H-4), 2.10 (1H, m, H-14), 2.02 (1H, m, H-12), 1.99 (1H, m, H-2), 1.91 (3H, d, J = 1.2 Hz, H-28), 1.85 (1H, m, H-2), 1.78 (1H, m, H-16), 1.74 (3H, d, J = 1.1 Hz, H-29), 1.74 (1H, m, H-9), 1.72 (1H, m, H-11), 1.59 (1H, m, H-15), 1.58 (1H, m, H-11), 1.52 (1H, m, H-15), 1.39 (1H, m, H-12), 1.38 (6H, s, H-26, H-27), 1.38 (1H, m, H-17), 1.29 (1H, m, H-16), 1.00 (1H, d, J = 6.6 Hz, H-21), 0.59 (3H, s, H-18). **^13^C NMR** δ [ppm]: 153.9 (C-10), 144.9 (C-8), 139.1 (C-24), 138.0 (C-23), 135.4 (C-5), 135.2 (C-24a), 132.6 (C-22), 123.3 (C-6), 117.4 (C-7), 109.2 (C-19), 71.7 (C-25), 71.3 (C-1), 66.5 (C-3), 58.2 (C-17), 57.7 (C-14), 46.9 (C-13), 42.9 (C-2), 41.8 (C-12), 37.5 (C-4), 36.8 (C-20), 31.3 (C-26 or C-27), 31.1 (C-26 or C-27), 30.0 (C-9), 28.7 (C-16), 24.6 (C-11), 23.4 (C-29), 23.2 (C-15), 21.8 (C-21), 17.0 (C-28), 12.9 (C-18). **UV** (CH_3_CN:H_2_O 1:1): λ_max_ = 274.8 nm. **HRMS** (TOF ES^+^) calc’d for C_30_H_46_O_3_K [M+K]^+^ 493.3084, found 493.3088. **PRI-1938**: ^1^H NMR δ [ppm]: 6.55 (1H, d, J = 11.5 Hz, H-6), 5.93 (1H, d, J = 11.5 Hz, H-7), 5.61 (1H, s, H-24a), 5.37 (1H, d, J = 9.7 Hz, H-22), 5.06 (1H, s, H-19), 4.96 (1H, s, H-19), 4.44 (1H, m, H-1), 4.13 (1H, m, H-3), 2.91 (1H, m, H-9), 2.73 (1H, m, H-4), 2.54 (1H, m, H-20), 2.27 (1H, m, H-4), 2.12 (1H, m, H-14), 2.05 (1H, m, H-12), 2.00 (1H, m, H-2), 2.00 (3H, s, H-28), 1.83 (1H, m, H-2), 1.80 (1H, m, H-16), 1.79 (3H, s, H-29), 1.75 (1H, m, H-9), 1.74 (1H, m, H-11), 1.62 (1H, m, H-15), 1.61 (1H, m, H-11), 1.54 (1H, m, H-15), 1.50 (1H, m, H-17), 1.43 (1H, m, H-12), 1.40 (6H, s, H-26, H-27), 1.24 (1H, m, H-16), 1.03, (1H, d, J = 6.5 Hz, H-21), 0.65 (3H, s, H-18). **^13^C NMR** δ [ppm]: 153.9 (C-10), 144.8 (C-8), 139.9 (C-24), 135.5 (C-23), 135.5 (C-5), 134.2 (C-22), 133.8 (C-24a), 123.3 (C-6), 117.4 (C-7), 109.3 (C-19), 71.6 (C-25), 71.4 (C-1), 66.5 (C-3), 58.7 (C-17), 57.7 (C-14), 47.0 (C-13), 42.9 (C-2), 41.7 (C-12), 37.5 (C-4), 36.8 (C-20), 31.4 (C-26 or C-27), 31.3 (C-26 or C-27), 30.0 (C-9), 28.3 (C-16), 24.6 (C-11), 23.2 (C-15), 21.1 (C-21), 15.4 (C-28), 15.0 (C-29), 12.9 (C-18). **UV** (CH_3_CN:H_2_O 1:1): λ_max_ = 241.8 and 273.6 nm. **HRMS** (TOF ES^+^) calc’d for C_30_H_46_O_3_K [M+K]^+^ 493.3084, found 493.3078.

#### 2.2.6. (1S,3R,5Z,7E,22Z,24E)-24-Dehydro-24a-homo-23-methyl-1,25-dihydroxyergocalciferol (**PRI-1927**)

**PRI-1927** was obtained as described in Section 2.2.4. and Section 2.2.5. for the synthesis of **PRI-1937** and **PRI-1938**; however, it was the only isolated vitamin D product. **^1^H NMR** δ [ppm]: 6.34 (1H, d, J = 11.2 Hz, H-6), 6.10 (1H, d, J = 11.2 Hz, H-7), 5.31 (1H, m, J = 1.7 Hz, H-19), 5.28 (1H, d, J = 1.2 Hz, H-24a), 4.92 (1H, m, H-19), 4.88 (1H, dd (overlaps with OH of solvent), H-22), 4.37 (1H, m, H-1), 4.15 (1H, m, H-3), 2.88 (1H, m, H-9), 2.54 (1H, m, H-4), 2.48 (1H, m, H-20), 2.28 (1H, m, H-4), 2.04 (1H, m, H-14), 2.01 (1H, m, H-12), 1.91 (2H, m, H-2), 1.91 (3H, d, J = 1.2 Hz, H-28), 1.74 (3H, d, J = 1.2 Hz, H-29), 1.73 (1H, m, H-16), 1.71 (1H, m, H-9), 1.70 (1H, m, H-11), 1.57 (1H, m, H-11), 1.51 (1H, m, H-15), 1.43 (1H, m, H-15), 1.38 (3H, s, H-26 or H-27), 1.37 (3H, s, H-26 or H-27), 1.37 (1H, m, H-12), 1.35 (1H, m, H-17), 1.24 (1H, m, H-16), 0.99 (1H, d, J = 6.6 Hz, H-21), 0.57 (3H, s, H-18). **^13^C NMR** δ [ppm]: 149.8 (C-10), 142.5 (C-8), 139.1 (C-23), 138.0 (C-24), 135.7 (C-5), 135.2 (C-24a), 132.6 (C-22), 124.9 (C-6), 118.9 (C-7), 112.1 (C-19), 71.7 (C-25), 71.5 (C-1), 67.4 (C-3), 58.1 (C-17), 57.6 (C-14), 46.9 (C-13), 46.2 (C-4), 43.7 (C-2), 41.8 (C-12), 36.8 (C-20), 31.3 (C-26 or C-27), 31.1 (C-26 or C-27), 30.0 (C-9), 28.7 (C-16), 24.6 (C-11), 23.4 (C-29), 23.2 (C-15), 21.8 (C-21), 17.0 (C-28), 12.8 (C-18). **UV** (CH_3_CN:H_2_O 1:1): λ_max_ = 266.5 nm. **HRMS** (TOF ES^+^) calc’d for C_30_H_46_O_3_Na [M+Na]^+^ 477.3345, found 477.3347.

### 2.3. Metabolic Resistance of Vitamin D Analogs to hCYP24A1- and hCYP3A4-Mediated Degradation

The CYP24A1-mediated degradation of vitamin D analogs was examined using recombinant *h*CYP24A1, as described previously [7,11]. The reaction mixture containing 2.0 μM bovine adrenodoxin, 0.2 μM bovine adrenodoxin reductase, 20 nM *h*CYP24A1, 5 μM vitamin D analog, 1mM NADPH, 100 mM Tris-HCl (pH 7.4), and 1mM EDTA in a total volume of 100 μL was incubated at 37 °C for 15 min. For *h*CYP3A4-mediated degradation, commercially available recombinant *h*CYP3A4 microsomes expressed in Sf-9 insect cells using the baculovirus expression system Corning Inc., New York, NY, USA, was used. The reaction mixture contained 20 nM *h*CYP3A4, 1 mM NADPH, 5 μM of each vitamin D analog, and 100 mM potassium phosphate buffer (pH 7.5), and was incubated at 37 °C for 15 min. The *h*CYP24A1 and *h*CYP3A4 reactions were terminated by adding 4 volumes of chloroform/methanol (3:1) under vigorous shaking. The organic phase was retrieved and dried. The resultant residue was dissolved in acetonitrile and centrifuged at 20,000× *g* for 15 min. The supernatant 20 μL sample was analyzed by HPLC as previously described [8]. The amount of analogs and metabolites detected in the eluates was calculated from the HPLC peak area. The percentage of metabolic conversion of a vitamin D analog was calculated as the ratio of the peak area of metabolites to the sum of the peak areas of the remaining vitamin D analog and the metabolite or metabolites (assumed as 100%).

### 2.4. Theoretical Calculations

#### 2.4.1. Preparation of Vitamin D_3_ Analogs and Structural Models of *r*CYP24A1 and *h*CYP3A4 Enzymes

**PRI-1906**, **PRI-1927**, **PRI-1937**, and **PRI-1938** were analyzed in their neutral form. The starting structures were generated using the Discovery Studio v. 22.1 visual interface by BIOVIA [15]. The molecular structures of all compounds were optimized using density functional theory (DFT) with the B3LYP/6-311G(d,p) hybrid functional, as implemented in Gaussian16 [16]. ESP atomic partial charges for all atoms were computed using the Breneman model to reproduce the molecular ESP [17]. The crystal structures of recombinant rat CYP24A1 (PDB ID: 3K9V) and human CYP3A4 (PDB ID: 2V0M) were obtained from the Brookhaven Protein Data Bank (https://www.rcsb.org/, accessed on 15 July 2025). The resolutions of these files were 2.5 and 2.8 Å for rCYP24A1 and hCYP3A4, respectively. Since no experimentally determined crystal structure of human CYP24A1 (*h*CYP24A1) is currently available in the Protein Data Bank, the rat CYP24A1 structure (3K9V) was used as a structural model for molecular docking and molecular dynamics simulations. This structure is commonly reported due to its high sequence identity (~85%) with *h*CYP24A1 and the conservation of critical residues within the active site [18]. Therefore, it represents the most accurate available template for structural and computational analysis of CYP24A1–ligand interactions. The PDB files contain co-crystals with 3-[(3-cholamidopropyl)dimethylammonio]-1-propanesulfonate (CHAPS) in the active site of *r*CYP24A1 and ketoconazole in the active site of CYP3A4. All ligands (except for the heme group), inorganic ions, and solvent molecules present in the original enzyme structures were manually removed, and hydrogen atoms were added to reflect physiological pH using the graphical interface of Discovery Studio v. 22.1. Prior to analysis, the iron atom in the heme group was constrained as Fe^2+^ to preserve its bonding to the nitrogen atoms of the heme after applying the CHARMm force field.

#### 2.4.2. Initial System Configurations: Molecular Docking

Molecular docking was used to identify potential binding sites and modes of interaction between the studied molecules and the enzymes (*r*CYP24A1 and *h*CYP3A4). Docking studies were conducted using GOLD Hermes 2024.3.0 (Genetic Optimization for Ligand Docking) [19,20,21], which employs a genetic algorithm to explore ligand conformational space while allowing for partial flexibility of the receptor, particularly within the binding site. The active site of each enzyme was defined based on the co-crystallized substrate or inhibitor positions in the crystal structures obtained from the RCSB Protein Data Bank. Four GOLD scoring functions—ChemScore, ChemPLP, GoldScore, and ASP—were evaluated in terms of their ability to correctly reproduce known binding poses and rank ligands effectively. Among these, ChemPLP is often favored for its ability to model hydrogen bonding and van der Waals interactions with high accuracy, while ASP accounts for solvation effects. Redocking of the co-crystallized reference ligands was conducted to validate the docking protocol. The resulting docking poses were compared to the original crystallized reference ligands to calculate the root-mean-square deviation (RMSD). Only docking solutions with RMSD < 2.0 Å and high consensus scoring across multiple functions were considered reliable for further analysis. The obtained RMSD values of 0.543 Å for *r*CYP24A1 and 0.463 Å for *h*CYP3A4 confirmed the validity of the applied GOLD ChemPLP protocol. Additionally, the docking results provided input geometries for molecular dynamics (MD) simulations, allowing refinement of binding poses and assessment of **PRI-1906**, **PRI-1927**, **PRI-1937**, and **PRI-1938** stability within the active site over time.

#### 2.4.3. Interaction with Enzyme Models *r*CYP24A1 and *h*CYP3A4: Molecular Dynamics Simulations

MD simulations provide insight into conformational and molecular behavior at the atomic level, as well as molecular motions, functions, and enzyme mechanisms [22]. It additionally assesses the stabilities of the final complex compounds used as initial structures for simulations conducted over 100 ns. This is achieved by first applying the CHARMM force field to both the enzyme and small molecules. The enzyme–ligand systems were solvated for the simulation by adding sufficient water molecules (TIP3P model) to enable free interaction between the enzyme and the solvent, allowing the simulation to run with periodic boundary conditions to prevent surface artifacts [23,24]. The starting structures of each system were immersed in a rectangular TIP3P water box, ensuring that the receptor atom was at least 10 Å away from the nearest edge of the box. The system components were neutralized by adding Na^+^ and Cl^−^ ions [25]. The MD simulations in Discovery Studio v. 22.1 were conducted using the Standard Dynamic Cascade technique (Minimization, Heating, Equilibration, and Production). Energy minimizations were performed using the steepest descent algorithm for 3000 steps, followed by the conjugate gradient algorithm for 4000 steps to reduce unfavorable intermolecular steric contacts (until the RMS gradient of the structure was below 0.01 (kcal/mol)/Å). The details of the MD protocol were as follows: a heating step was performed for 50 ps with a time step of 1 fs, during which the system was heated from 50 K to 300 K; before the production stage, the system was equilibrated by allowing it to evolve spontaneously until the average temperature and structure remained stable and the total energy converged; the total duration of the dynamic simulation was set to 100 ps; the equilibrated system served as the starting structure for 100 ns production runs in the NPT ensemble at a temperature of 300 K using a Berendsen thermostat [26]; coordinates were recorded every 10 ps, and trajectory structures for analysis were saved every 1.2 ns. All energy minimization and MD simulations were performed using the Particle Mesh Ewald (PME) algorithm [27], which accounts for the proper treatment of electrostatic interactions [28].

#### 2.4.4. Evaluating Binding Free Energy with Molecular Mechanics Poisson–Boltzmann Surface Area (MM/PBSA)

Although molecular docking combined with MD simulations provides a clear image of the shape complementarity between the ligand and the enzyme, additional essential information regarding the binding free energy (ΔG*_bind_*) is required. ΔG*_bind_* of the enzyme–ligand complexes was computed using molecular mechanics/Poisson–Boltzmann surface area (MM/PBSA) analysis performed with adaptive Poisson–Boltzmann Discovery Studio v. 22.1 [29]. The MM/PBSA approach is one of the most used techniques for computing interaction energies within biomolecular complexes [30,31,32,33]. Combined with MD simulations, MM/PBSA can effectively interpret significant structural changes and contributions to entropic binding energy. These calculations were conducted using the last 200 frames and were determined by applying the following equation: ΔG*_bind_* = ΔG_(enzyme-ligand)_ − ΔG_enzyme_ − ΔG_ligand_, where ΔG_(enzyme-ligand)_ represents the total free energy of the enzyme–ligand complex, and ΔG_enzyme_ and ΔG_ligand_ are the total free energies of the separated enzyme and ligand, respectively.

## 3. Results and Discussion

### 3.1. Synthesis of Analogs **PRI-1927**, **PRI-1937**, and **PRI-1938**

Previously, we have shown that a side-chain extended, and branched analog of vitamin D, **PRI-1906**, containing a methyl substituent 28-CH_3_ on carbon C-24 (Figure 1), exhibits an antiproliferative activity several times higher against the human breast cancer lines T47D and MCF7, as well as the human and mouse leukemia cell lines HL-60 and WEHI-3, than that of 1,25D3. To test the extent of this trend, we conceived **PRI-1927** with an extra 29-CH_3_ on C-23 of the natural 5,6-*cis* (5*Z*) triene system geometry (Figure 2). Vitamin D analogs with a 5,6-*trans* (5*E*) configuration of the triene system are more active than analogs with a natural 5,6-*cis* (5*Z*) configuration. For this reason, we also designed analogs **PRI-1937** and **PRI-1938** containing a 5,6-*trans* configuration.

**Scheme 1:**
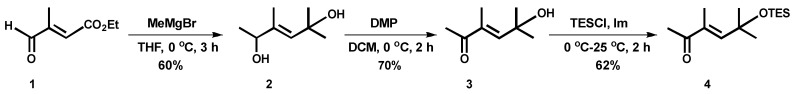
Synthesis of the side chain ketone **4**.

For the synthesis of new analogs, we prepared side-chain ketone **4** (Scheme 1) from the known [10] aldehyde **1**. Initially, **1** was reacted with a Grignard reagent (MeMgBr), yielding diol **2** in 60% yield. Next, diol **2** was chemoselectively oxidized to ketone **3** with Dess-Martin periodinane (DMP) in 70% yield. Ketone **3** was protected with triethylsilyl chloride (TES-Cl), resulting in the final side-chain ketone **4** in 62% yield.

**Scheme 2:**
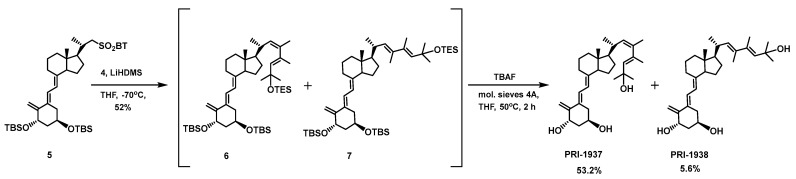
Synthesis of the new analogs of 1,25D3: **PRI-1937** and **PRI-1938**.

Previously, using vitamin D C-22 phenyl sulfone [10] or benzothiazole sulfone [34] in combination with side-chain aldehydes, or using vitamin D C-22 aldehyde [2] with side-chain phenyl sulfone, we obtained exclusively 22-*trans* coupling products via classical Julia olefination. Surprisingly, the coupling of benzothiazole **5** with side-chain ketone **4,** followed by desilylation, afforded mainly 22-*cis* analog **PRI-1937** (in 53.2% yield) and 22-*trans* analog **PRI-1938** only as a minor product (in 5.6% yield) (Scheme 2). In contrast, **PRI-1927** was the only product isolated from the coupling of ketone **4** with 5,6-*cis* benzothiazole, followed by desilylation. Coupling the side chain ketone **4** via a modified Julia olefination with C-22 benzothiazole sulfone [35] **5** gave a mixture of 22-*cis* and 22-*trans* olefines **6** and **7**, respectively (Scheme 2) in a total 52% yield. This mixture was used for the next step without separation of the isomeric olefins. The availability of **PRI-1938** enabled a more direct comparison with **PRI-1906**, which also features an all-*trans*-22,24 side chain.

**Scheme 3:**
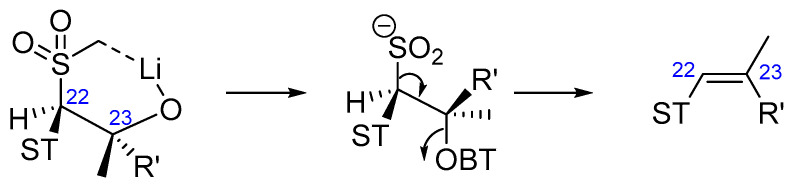
Proposed conversion of the transition state to 22-*cis* analogs **PRI-1927** and **PRI-1937** (ST—steroid framework and BT—benzothiazole).

The one-pot Julia olefination of heteroaryl sulfone does not proceed through the equilibrating intermediates. The geometry of the resulting olefin depends on the structure of the carbonyl substrate. This olefination usually gives a mixture of geometric isomers containing mainly 22-*trans* olefin [34] and, in some cases, 22-*cis* olefin as a byproduct. However, coupling the α-methyl ketone **4** with benzothiazole sulfone **5** leads, after tetrabutylammonium fluoride desilylation, mainly 22-*cis* olefin **PRI-1937** and 22-*trans* olefin **PRI-1938** (Scheme 2). According to quantum chemical calculations, **PRI-1938** is thermodynamically more stable than **PRI-1937**. This is consistent with its gradual formation upon prolonged standing of **PRI-1937** in the solid state at 5 °C. The mixture of **PRI-1937** and **PRI-1938** was separated using preparative reverse-phase HPLC. Interestingly, the 5,6-*cis*-22-*cis* isomer **PRI-1927** did not isomerize to the respective 5,6-*cis*-22-*trans* analog (not shown). The UV spectrum of **PRI-1938** (Figure S1) shows an absorption maximum of 273.6 nm, characteristic of 5,6-*trans* vitamin D analogs [34], and a maximum at 241.8 nm, typical for the side chain of all-*trans* conjugated diene [10]. Unexpectedly, the UV spectrum of **PRI-1937** (Figure S2), with the *cis*-*trans*-conjugated diene in the side chain, shows the absorption maximum only at 274.8 nm, characteristic of 5,6-*trans* vitamin D analogs, but no additional maximum at around 240 nm. This single maximum might result from the two overlapping maxima, originating from the triene system and the isomerized side-chain diene. As expected, **PRI-1927** shows an absorption maximum (Figure S3) at 266.5 nm, characteristic of 5,6-*cis* vitamin D analogs [34]. In the modified Julia olefination (Scheme 3), the favored formation of the *cis* product may result from the kinetically formed Li-chelate transition state [35] and an anti-periplanar elimination pathway. This is due to minimized steric strain and optimal orbital overlap in the intermediate structure.

### 3.2. Structure Assignment of **PRI-1927**, **PRI-1937,** and **PRI-1938** by TOF MS ES^+^ and ^1^H and ^13^C NMR

**Figure 3:**
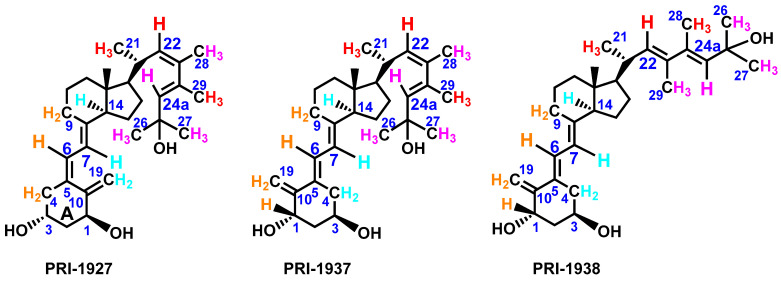
Structures of **PRI-1927**, **PRI-1937**, and **PRI-1938** showing NOE assignments in ^1^H NMR spectra for characteristic protons of the side chain and the A-ring in colors.

The MS TOF ES^+^ showed the identical fragmentation pattern for **PRI-1937** and **PRI-1938**, indicating that the transformation product **PRI-1938** is an isomer of **PRI-1937**. Both spectra showed the same characteristic signals at *m*/*z* 493 (M+39) and 477 (493-H-CH_3_). The high-resolution mass spectrum for these signals agrees well with the calculated data. All signals were assigned in the ^1^H and ^13^C NMR spectra (Figures S4–S9) to the respective proton and carbon atoms. Both 1D and 2D NMR techniques (COSY, HSQC, and HMBC) unambiguously confirmed the chemical structures of **PRI-1927**, **PRI-1937**, and **PRI-1938**. The NOESY sequences identified the spatial relationship between side chain protons in the geometric isomers **PRI-1937** and **PRI-1938** and the A-ring protons of **PRI-1927**. The spatial NOE interactions were mapped based on the chemical shifts of key protons in the NOESY spectra, revealing the relative spatial proximity of specific protons. Correlation signals for **PRI-1938** showed that the H-22 proton (Figure 3, marked in red) is close to the H-21 protons (red) and H-28 protons (red). The H-24a proton (magenta) is near the H-29 protons (magenta) and the H-26/27 protons (magenta). These data confirmed the geometry of the double bonds in the side chain of **PRI-1938** as (22*E*,24*E*). The H-22 proton (red) of **PRI-1937** is close to the H-21 and H-29 protons (red). The H-24a proton (magenta) is near the H-26/27 protons (magenta), and the H-28 protons (magenta) are spatially adjacent to the H-29 protons (red) and H-26/27 protons (magenta). This arrangement indicates the (22*Z*,24*E*) side chain configuration of **PRI-1937**. The proton interactions in the side chain of **PRI-1927** were like those for **PRI-1937**, confirming that **PRI-1927** also has the (22*Z*,24*E*) geometry. Proton correlations determined the configuration of the triene system in all analogs. The H-6 proton (orange) in the spectrum of **PRI-1937** and **PRI-1938** shows correlations with the H-9 and H-19 protons (orange). The H-19 protons (orange) correlate with the H-1 proton (orange), and the H-7 proton (light blue) shows correlations with the H-10 and H-14 protons (light blue). Thus, the 5,6-*trans* (5*E*) geometry was assigned to the triene system of **PRI-1937** and **PRI-1938**. The proton’s correlation is different for **PRI-1927**. In its spectrum, the H-6 proton (orange) correlates with the H-9 and H-4 protons (orange), and the H-7 proton (light blue) correlates with the H-14 and H-19 protons (light blue). These correlations led to the assignment of the 5,6-*cis* (5*Z*) geometry to the triene system of **PRI-1927**.

### 3.3. Metabolic Conversion of 1,25D3 and Its Analogs by Cytochrome CYP3A4

**Figure 4:**
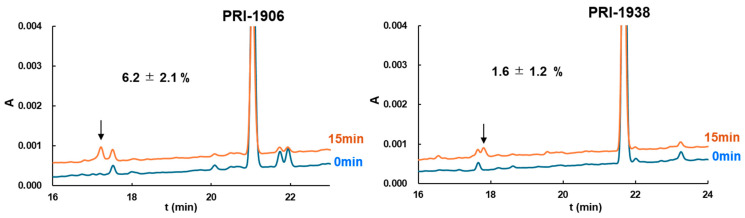
HPLC profiles of analogs **PRI-1906** and **PRI-1938** and their metabolites generated by *h*CYP3A4. The peaks marked with arrows indicate putative metabolites.

The metabolic conversion of the 22,24-all-trans analog **PRI-1938** (Figure 4, 1.6%) was more than nine times lower than that of 1,25D3 (Figure S10 and Table 1, 14.9%). The comparable metabolic conversion of both 22-cis-24-trans analogs, **PRI-1927** and **PRI-1937** (Figure S10, 16.7% and 16.3%, respectively), against nonselective CYP3A4 was similar to that of 1,25D3 (14.9%). The conversion of **PRI-1906** (Figure 4) and **PRI-1938** was 6.2% and 1.6%, respectively. This corresponds to approximately a four-fold lower conversion rate for **PRI-1938**, indicating enhanced metabolic stability compared to **PRI-1906**. The structural difference between **PRI-1938** and **PRI-1906** is present in both parts of the vitamin D structure responsible for activity.

Both analogs have the rigid all-*trans* geometry of the side chain, but **PRI-1938** contains an additional C-29 methyl attached to C-23. Additionally, **PRI-1938** has the 5,6-*trans* geometry of the triene system in contrast to the natural 5,6-*cis* geometry in **PRI-1906**. The metabolic conversion of **PRI-1927** (16.7%) is comparable to **PRI-1937** (16.3%) and considerably higher than that of **PRI-1906** and **PRI-1938**. This means that for the 22-*cis*-24-*trans* analogs, the side chain geometry, but not the triene system geometry, influences the decreased resistance to *h*CYP3A4 metabolism.

### 3.4. Metabolic Conversion of 1,25D3 and Its Analogs by Cytochrome CYP24A1

**Figure 5:**
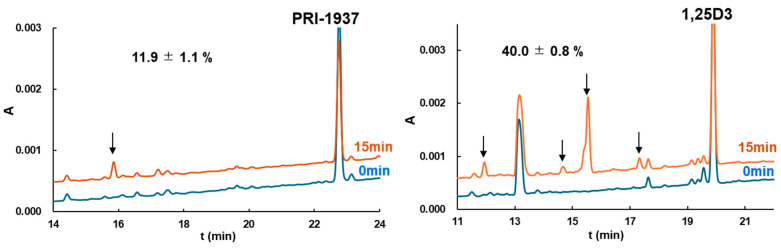
HPLC profiles of analogs **PRI-1937** and 1,25D3 and their metabolites generated by *h*CYP24A1. The peaks marked with arrows indicate putative metabolites.

The metabolic conversion of the novel analog **PRI-1938** (Figure S11), at 5.2%, was comparable to that of the previously characterized **PRI-1906** (4.4%), indicating similar biotransformation profiles in the studied system. For the new 22-*cis*-24-*trans* analogs, **PRI-1927** and **PRI-1937** (Figure S11 and Figure 5), metabolic conversion rates (14.2% and 11.9%, respectively; see Table 1) were closely aligned but more than 2-fold higher than that of the 22,24-all-*trans* analog **PRI-1938**. These findings suggest that stereochemical modifications at positions 22 and 24 significantly influence the susceptibility of these molecules to metabolic transformation. Notably, **PRI-1938** exhibited nearly 8-fold lower metabolic conversion compared to 1,25D3, indicating its increased resistance to CYP24A1-mediated catabolism and its potential for applications requiring prolonged biological activity. Further studies, including enzymatic kinetic analyses and characterization of specific metabolic pathways, are warranted to elucidate the mechanisms underlying these observed differences.

### 3.5. Characterization of the Binding Sites for Vitamin D_3_ Analogs in the CYP24A1 and hCYP3A4 Models by Molecular Docking

**Figure 6:**
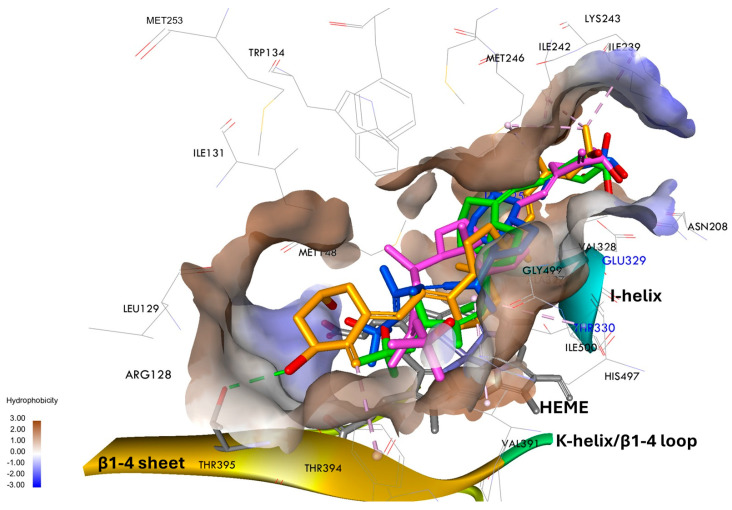
Close-up view of the active site of the rat CYP24A1 structure after molecular docking. Binding of 1,25D3 analogs (**PRI-1906**, **PRI-1927**, **PRI-1937**, and **PRI-1938**) in the active site of *r*CYP24A1. **PRI-1906** (C atoms shown as orange), **PRI-1927** (C atoms shown as green), **PRI-1937** (C atoms shown as pink), and **PRI-1938** (C atoms shown as blue). Surface hydrophobicity is depicted by shaded colors: negative values (blue) correspond to hydrophilic residues, while positive values (brown) correspond to hydrophobic residues.

Vitamin D_3_ metabolism in humans involves multiple cytochrome P450 enzymes, with *h*CYP3A4, the primary hepatic isoform, and CYP24A1, a key mitochondrial enzyme in target tissues, playing critical roles in hydroxylation and catabolism, respectively. *h*CYP3A4 deactivates both native 1,25D3 and its synthetic analogs, while CYP24A1 specifically regulates vitamin D catabolism. Understanding the interactions of novel vitamin D analogs with these enzymes is crucial for predicting their metabolic stability and optimizing pharmacological profiles. To elucidate the molecular basis for the differential metabolic stability of analogs (**PRI-1906**, **PRI-1927**, **PRI-1937**, and **PRI-1938**), molecular docking studies were conducted with *r*CYP24A1 and *h*CYP3A4. All compounds accessed the active site of *r*CYP24A1 via the vitamin D access tunnel, interacting with multiple residues (Arg128, Leu129, Ile131, Trp134, Met148, Ile242, Lys243, Met246, Phe249, Leu325, Ala326, Val328, Glu329, Val391, Phe393, Ile500, and heme group (HEM520)). These residues are known to form various interactions with 1,25D3 (Figure 6 and Figure S12) [14,36,37]. The open conformation of *r*CYP24A1 is structurally favorable for the binding of 1,25D3, with key amino acid residues mediating substrate recognition and orientation. Notably, Glu329 plays a critical role in stabilizing the substrate within the active site through electrostatic interactions, contributing to high-affinity binding. Additionally, residues such as Arg128, Met246, Ala326, and Ile500 participate in shaping the substrate-binding pocket and influence the regioselectivity of hydroxylation by affecting substrate positioning relative to the heme group. All of the analogs bind within the hydrophobic pocket of *r*CYP24A1, adopting similar orientations, with the CD-rings positioned above the heme group (Figure 6). This binding mode corresponds to the specificity of the enzyme’s active site, which is optimally configured to accommodate the steroidal scaffold of vitamin D analogs. However, a detailed analysis of the ligand positions revealed significant differences in the orientation of some analogs, which may influence their binding affinity and susceptibility to metabolism.

The compounds **PRI-1906** and **PRI-1938** enter the pocket in an orientation similar to that of 1,25D3, positioning their side chains near the Glu329 residue, which is crucial for high-affinity substrate binding (Figure 6). This orientation promotes strong interactions with the active site and may hinder enzymatic access to hydroxylation sites, potentially explaining the high metabolic resistance of **PRI-1938** (5.2%). On the other hand, **PRI-1927** and **PRI-1937** adopt an inverted orientation, directing their side chains toward the entrance of the binding pocket (Figure 6). In this position, they can interact with the Ala326 and Ile500 residues, which, according to previous studies, influence the regioselectivity of hydroxylation by *r*CYP24A1. This binding mode is less favorable for high affinity, as reflected by their greater susceptibility to metabolism (14.2%). The observed differences in ligand orientation correlate well with the experimental results and confirm the relationship between binding mode, binding affinity, and the degree of metabolic conversion of the tested analogs.

**Figure 7:**
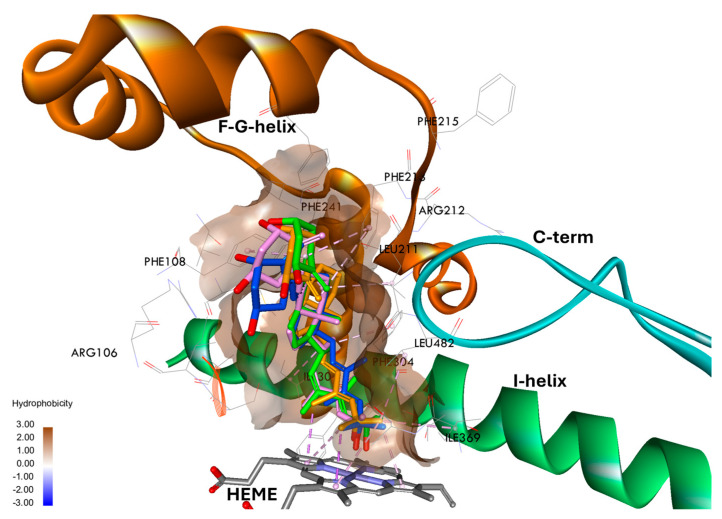
Close-up view of the active site of the human CYP3A4 structure after molecular docking. Binding of 1,25D3 analogs (**PRI-1906**, **PRI-1927**, **PRI-1937**, and **PRI-1938**) in the active site of *h*CYP3A4. **PRI-1906** (C atoms shown as orange), **PRI-1927** (C atoms shown as green), **PRI-1937** (C atoms shown as pink), and **PRI-1938** (C atoms shown as blue). Surface hydrophobicity is depicted by shaded colors: negative values (blue) correspond to hydrophilic residues, while positive values (brown) correspond to hydrophobic residues.

In contrast to *r*CYP24A1, where ligand orientation within the active site varied considerably and correlated with metabolic stability, all tested compounds adopted a similar binding mode in *h*CYP3A4, entering the binding pocket with their side chain directed toward the heme group (Figure 7). This uniform orientation reflects the broader and more flexible nature of the *h*CYP3A4 active site, which can accommodate structurally diverse ligands in comparable poses. Despite these architectural and recognition differences between the two enzymes, their experimental metabolic conversion rates were similar. This suggests that, while *r*CYP24A1 requires a specific ligand orientation and defined interactions with key residues for efficient catalysis, *h*CYP3A4 primarily depends on side-chain accessibility to the heme center, highlighting distinct mechanisms of substrate recognition and hydroxylation. The three-dimensional structure of the *h*CYP3A4 active site (Figure S13), based on the crystal structure with ketoconazole (PDB ID: 2V0M), reveals a large, predominantly hydrophobic binding pocket formed by residues Phe57, Arg106, Phe108, Ile120, Ser119, Leu211, Phe213, Ile301, Phe304, Ala305, Ile369, Ala370, Met371, Arg372, Glu374, Thr309, and Leu482. Key hydrophobic residues, including Phe304, Ile301, Ile369, and Ala305, form a stabilizing cluster near the heme group, facilitating effective ligand binding and catalytic interactions. Additionally, polar residues such as Ser119 and Thr309 participate in hydrogen bonding, enhancing affinity for ligands capable of polar interactions, as demonstrated in the ketoconazole-bound structure.

Unlike *r*CYP24A1, where residues such as Glu329, Ala326, and Ile500 play crucial roles in substrate binding and regioselectivity during hydroxylation of analogs, *h*CYP3A4 exhibits greater conformational adaptability, enabled by its expansive binding site (~1380 Å^3^). Ligand positioning in *h*CYP3A4, as illustrated in the 2V0M structure, is primarily determined by steric complementarity and proximity to the heme iron, with contributions from both hydrophobic and polar contacts. This structural versatility enables consistent binding modes for structurally related compounds, including 1,25D3 analogs, as confirmed by molecular docking studies using the ChemPLP scoring function. To assess the stability of the tested analogs within the active sites of *r*CYP24A1 and *h*CYP3A4, MD simulations were performed for both the *r*CYP24A1–ligand and *h*CYP3A4–ligand complexes.

### 3.6. Binding Free Energy and Interactions of Vitamin D_3_ Analogs with rCYP24A1

MD simulations were conducted to investigate the binding modes of 1,25D3 analogs within the active sites of *r*CYP24A1 and *h*CYP3A4, with particular attention to the role of the I-helix and the heme moiety in stabilizing ligand interactions and influencing catalytic efficiency. The root-mean-square deviation (RMSD) values between the initial docking poses and the equilibrated MD structures remained consistently low (<2 Å) for all compounds, indicating stable binding conformations. As anticipated, a complete transition to a catalytically closed state was not observed, consistent with the accessible simulation timescales. Nonetheless, the MD trajectories revealed notable local flexibility within the active site, particularly in regions critical for substrate recognition and orientation, such as the I-helix and the heme-proximal environment. Binding free energies for compounds **PRI-1906**, **PRI-1927**, **PRI-1937**, and **PRI-1938** in *r*CYP24A1, calculated using the MM-PBSA method, are presented in Table 1 alongside experimental metabolic stability data for both *r*CYP24A1 and *h*CYP3A4. For *r*CYP24A1, the binding affinities decrease in the order **PRI-1938** (−124.54 kcal/mol) > **PRI-1906** (−93.49 kcal/mol) > **PRI-1937** (−74.37 kcal/mol) > **PRI-1927** (−56.97 kcal/mol). This trend correlates well with experimental metabolic stability, where 22,24-all-*trans* analog **PRI-1938** exhibits approximately eight-fold greater resistance to *r*CYP24A1-mediated hydroxylation compared to the natural substrate, 1,25D3. This increased resistance likely stems from **PRI-1938’s** high-affinity binding and structural features that result in suboptimal positioning for heme-mediated catalysis, thereby hindering oxidative metabolism. As depicted in Figure 7, **PRI-1938** exhibited the most metabolically resistant binding pose (5.2% conversion) within the open conformation of *r*CYP24A1.

This conformation is stabilized by four key hydrogen bonds: the 3-hydroxyl group of the A-ring interacts with Arg128 and Leu129 in the B–B′ loop, while the 25-hydroxyl group of the side chain forms hydrogen bonds with Glu329 and Lys243. These interactions anchor the ligand in a position distal to the heme iron, thereby reducing its accessibility for catalytic turnover. In addition, the side chain of **PRI-1938** engages in favorable hydrophobic contacts: the 29-methyl interacts with Leu325 and Met246; the 28-methyl with Met246; and the 26,27-methyls with Met246, Ile242, and Lys243. The parallel orientation of the CD-rings promotes hydrophobic interactions with conserved residues Ala326, Met148, Trp134, and Val391, further stabilizing the ligand in a catalytically unfavorable orientation relative to the heme and the kinked region of the I-helix, which typically facilitates oxygen activation during hydroxylation. MD trajectories further revealed that **PRI-1938** exhibits altered positioning and reduced stability of side-chain interactions with the I-helix compared to 1,25D3, potentially disrupting optimal substrate alignment within the cylindrical active site and thereby further impairing catalytic efficiency. Similarly, **PRI-1906** adopts a comparable binding conformation within the open form of *r*CYP24A1, with its A-ring oriented to form strong hydrogen bonds via the 1- and 3-hydroxyl with Leu129, Arg128, and Met148. The presence of a 19-methylene at C-10 enhances hydrophobic interactions with Met148, further stabilizing the ligand–enzyme complex. The side chain of **PRI-1906** also engages in hydrophobic contacts: the 28-methyl interacts with Ile242, Leu325, and Val328, while the 26,27-dimethyl contacts Lys243, Ile242, and Val328. A key hydrogen bond between the 25-hydroxyl and Glu329, located near the I-helix, further stabilizes the complex and may contribute to suboptimal substrate positioning relative to the catalytic center. MD simulations revealed that, compared to the native ligand, **PRI-1906** exhibits reduced interaction stability with the I-helix, consistent with its high metabolic resistance (4.4% conversion), making it the second most stable analog evaluated. These localized perturbations within the active site, particularly in the regions surrounding the I-helix and heme, suggest that the structural modifications present in **PRI-1938** and **PRI-1906** impair catalytic turnover not only through steric hindrance—as observed in docking studies—but also by disrupting the dynamic features essential for efficient substrate recognition and catalysis.

**Figure 8:**
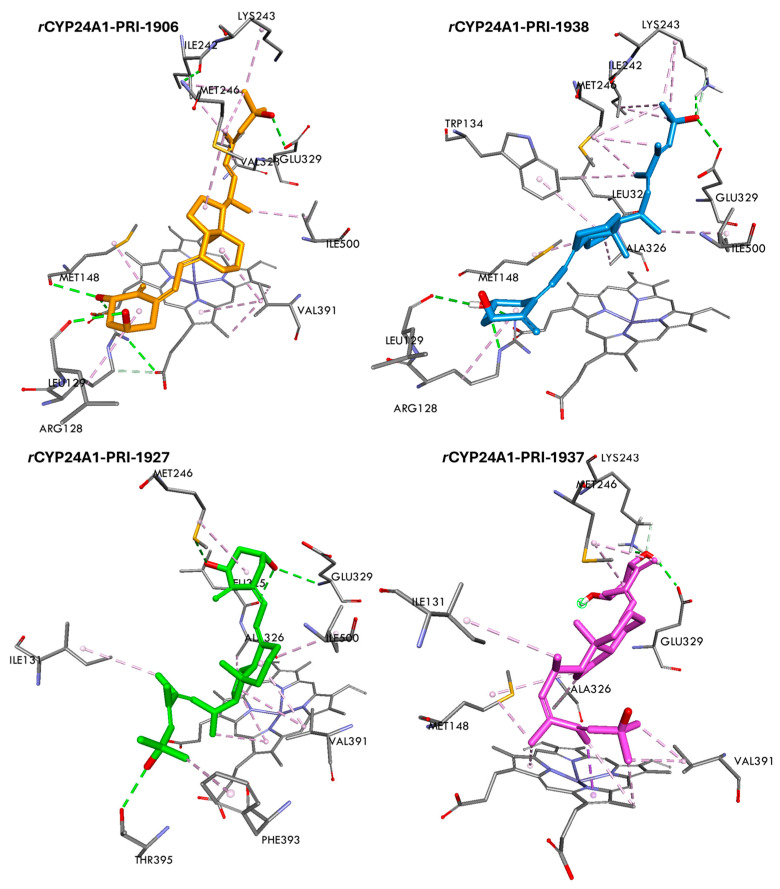
Molecular interactions of 1,25D3 analogs with *r*CYP24A1 from MD simulations. Magenta dashed lines indicate hydrophobic interactions; green lines indicate hydrogen bonds.

The 22-*cis*-24-*trans*, **PRI-1927** and **PRI-1937**, exhibit relatively high binding energies and low metabolic stability in their interactions with the *r*CYP24A1 active site, as evidenced by their conversion rates of 14.2% and 11.9%, respectively. These findings classify them as the least metabolically stable compounds among the tested analogs. Their unfavorable metabolic profiles can be attributed to specific structural and conformational features, including the positional shift of the 19-methylene from C-10 in **PRI-1927** to C-4 in **PRI-1937**, as well as the conformational constraints imposed by the 22-*cis*-24-*trans* geometry and a flexible U-shaped side chain (Figure 8). In **PRI-1937**, the enhanced binding affinity compared to **PRI-1927** results from distinct interactions within the substrate-binding cavity. The 3-hydroxyl of the A-ring forms hydrogen bonds with Lys243 and Glu329, anchoring the ligand in the polar region of the binding pocket. Concurrently, the 19-methylene at C-4 engages in stabilizing hydrophobic interactions with Met246. The U-shaped conformation of the side chain facilitates additional hydrophobic interactions with Met148 and enables π–π stacking and van der Waals contacts with the heme prosthetic group. While these interactions strengthen ligand binding, they also promote a conformation that positions reactive groups in proximity to the catalytic heme, thereby enhancing susceptibility to hydroxylation and explaining the low metabolic resistance of **PRI-1937**.

In contrast, **PRI-1927** establishes stabilizing hydrogen bonds via the 1- and 3-hydroxyl with Glu329, Leu325, and Met246 (Figure 8). Its 29-methyl contributes to hydrophobic stabilization through contacts with Ile131, while the CD-ring system engages in hydrophobic interactions with Ile500, Val391, and Ala326, positioning the core of the molecule near the heme center. However, the presence of the 19-methylene at C-10, in conjunction with the stereoelectronic constraints imposed by the 22-*cis*-24-*trans* configuration, restricts optimal hydrophobic interactions—particularly with Met246—thereby diminishing overall complex stability. This suboptimal orientation correlates with the compound’s elevated metabolic conversion rate, supporting the classification of **PRI-1927** as one of the least stable analogs in the series.

These findings underscore the critical role of the I-helix and the heme environment in modulating ligand binding and catalytic efficiency in *r*CYP24A1. The observed local disruptions in side chain dynamics and orientation relative to the I-helix provide mechanistic insights that extend beyond static docking results, supporting the hypothesis that specific structural modifications in 1,25D3 analogs reduce susceptibility to hydroxylation by altering both the static architecture and dynamic behavior of key active-site interactions.

### 3.7. Binding Free Energy and Interactions of Vitamin D_3_ Analogs with hCYP3A4

Binding free energies for analogs—**PRI-1938**, **PRI-1906**, **PRI-1937**, and **PRI-1927**—were calculated using MD simulations in combination with the MM-PBSA approach (Table 1). In the case of *h*CYP3A4, **PRI-1938** exhibited the highest binding affinity (−86.06 kcal/mol), followed by **PRI-1906** (−75.80 kcal/mol), **PRI-1937** (−70.61 kcal/mol), and **PRI-1927** (−51.80 kcal/mol).

**Figure 9:**
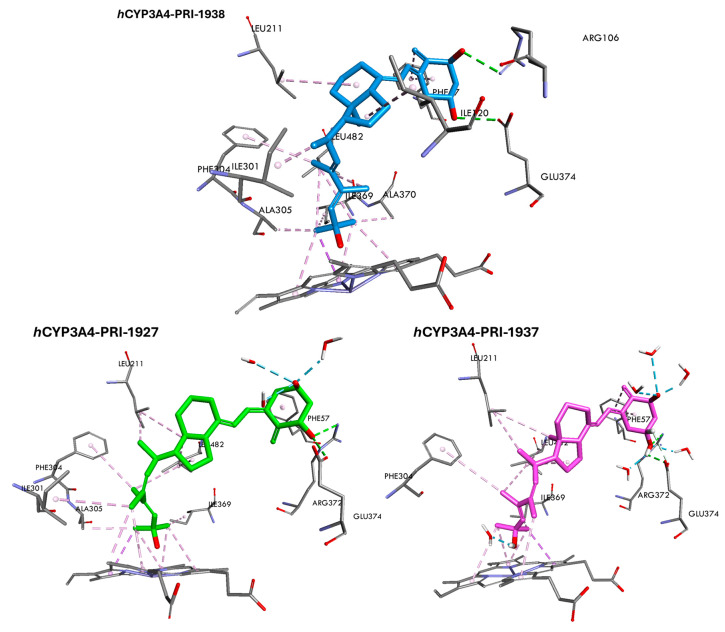
Molecular interactions of 1,25D3 analogs with *h*CYP3A4 from MD simulations. Magenta dashed lines indicate hydrophobic interactions; green lines indicate hydrogen bonds.

A parallel analysis for *r*CYP24A1 revealed a similar trend: **PRI-1938** demonstrated the strongest binding affinity and the highest metabolic resistance (1.6% conversion rate), whereas **PRI-1927** showed the weakest binding and the lowest metabolic resistance (16.7% conversion rate). These results indicate a strong inverse correlation between calculated binding free energies and metabolic conversion rates for both enzymes. The consistent structure–activity relationships observed across *h*CYP3A4 and *r*CYP24A1 suggest that specific structural features—such as A-ring hydroxylation patterns, side-chain modifications, and overall molecular geometry—play a decisive role in substrate recognition and metabolic stability. Despite differences in the active site architecture and physiological function of *h*CYP3A4 and *r*CYP24A1, these shared structure–function determinants appear to similarly influence ligand–enzyme interactions. This underscores their significance in the rational design of vitamin D_3_ analogs with improved metabolic resistance. The MD-generated orientations of ligands are presented in Figure 9.

**PRI-1938**, distinguished from **PRI-1906** by the 19-methylene at the C-4 and the presence of a 29-methyl, adopts a binding conformation within the *h*CYP3A4 active site comparable to that of **PRI-1906** [11]. In addition to conserved interactions, the side chain of **PRI-1938** engages in favorable hydrophobic contacts: the 29-methyl interacts with Phe304 and Leu482, while the 26,27-methyl forms hydrophobic interactions with Ala305, Ile369, Ala370, Leu482, and the heme prosthetic group (Figure 9). The A-ring 1- and 3-hydroxyl establish stabilizing hydrogen bonds with Asp76 and Glu374, further anchoring the ligand in the polar region of the binding pocket. Notably, the positioning of the 19-methylene at C-4 enables additional hydrophobic interactions with Phe57, while the CD-ring system contributes to binding stability through hydrophobic contacts with Ile120 and Leu211. In the context of *h*CYP3A4, **PRI-1927** and **PRI-1937** adopt comparable binding orientations within the enzyme’s active site. The 1-hydroxyl forms stable hydrogen bonds with Glu374 and Arg372, contributing to the anchoring of the ligand in the polar region of the binding cavity. Additionally, both the A- and C-rings participate in hydrophobic interactions with Phe57 and Leu482, respectively. The presence of a 19-methylene at the C-4 in **PRI-1937** further enhances hydrophobic contacts with Phe57, thereby contributing to the stabilization of the ligand within the active site. Notable differences between these analogs are observed in the interactions involving their side chains. In both compounds, the C28- and C29-methyl engage in hydrophobic interactions with Leu482, Phe304, and the heme group (Figure 9).

However, the 29-methyl in **PRI-1927** establishes an additional hydrophobic contact with Ile301, which is absent in **PRI-1937**. Furthermore, while the 26,27-methyls in both analogs interact hydrophobically with Ile369 and the heme moiety, **PRI-1927** forms supplementary hydrophobic contacts with Ala305, potentially contributing to subtle differences in binding affinity and orientation. Interestingly, both **PRI-1927** and **PRI-1937** are extensively solvated by water molecules within the *h*CYP3A4 binding pocket. This factor may influence their conformational stability and dynamic behavior during the binding process.

## 4. Conclusions

The modified Julia olefination of benzothiazole sulfones with aldehydes leads to a mixture of geometric isomers of *trans* olefins as the major products and *cis* olefins as byproducts. Unexpectedly, coupling of a vitamin D benzothiazole sulfone with an α-methyl ketone side-chain fragment afforded predominantly the 22-*cis* olefin (**PRI-1937**), with the *trans* olefin as a minor byproduct. This finding demonstrates that the structure of the carbonyl substrate directly influences the olefin’s geometry in this reaction system. The thermodynamically more stable 22,24-all-*trans* isomer **PRI-1938** was subsequently obtained via solid-state isomerization of **PRI-1937**. Interestingly, the 5,6-*cis*-22-*cis* analog **PRI-1927** did not undergo isomerization under the same conditions, highlighting the steric and conformational constraints imposed by A-ring modifications. Biological evaluation of these analogs revealed that both side chain and triene system modifications significantly affect their metabolic stability. Among the series, **PRI-1938** demonstrated the highest resistance to CYP3A4 and CYP24A1-mediated metabolism, while **PRI-1927** and **PRI-1937**, both 22-*cis*-24-*trans* isomers, exhibited comparable but markedly lower metabolic resistance. As anticipated for the broadly substrate-tolerant CYP3A4, the metabolic stability of the 22-*cis*-24-*trans* analogs resembled that of 1,25-dihydroxyvitamin D3. In contrast, the vitamin D-specific CYP24A1 displayed pronounced selectivity, with **PRI-1938** showing substantially enhanced resistance compared to the other analogs.

A comparison of the analogs **PRI-1937** and **PRI-1938** indicates a decisive influence of the side chain geometry on the metabolic stability of 1,25D3 analogs. These compounds differ solely in the geometry of their side chains. **PRI-1938**, with a 22,24-all-*trans* geometry—similar to that of **PRI-1906**—exhibits more than twice the metabolic stability of **PRI-1937**, which possesses a 22-*cis*-24-*trans* geometry. The impact of the triene system geometry on metabolic stability appears to be considerably smaller. The metabolic stability of **PRI-1927**, featuring a 5,6-*cis* triene configuration, is only slightly lower than that of **PRI-1937**, which has a 5,6-*trans* geometry. Therefore, in the design of new 1,25D3 analogs modified in the side chain, it is advantageous to retain the 22,24-all-*trans* geometry and to prefer the 5,6-*trans* geometry of the triene system. The effect of modifying the triene system by removing the 19-methylene group—typical of 19-*nor* analogs of 1,25D3—on metabolic stability remains to be elucidated. Moreover, it appears that determining the metabolic stability of a 1,25D3 analog serves as an appropriate preliminary criterion for selecting analogs for further studies of functional activity.

Additionally, it is reasonable to expect that **PRI-1938**, which is structurally similar to our previously synthesized VDR agonists, exerts its biological activity through binding to VDR. In the case of another potent VDR agonist, eldecalcitol, its functional effect of increasing bone mineral density is associated with a marked reduction in serum 1,25-dihydroxyvitamin D_3_ levels. This effect—potentially also relevant to **PRI-1938**—is presumed to result from analog-induced activation of VDR, leading to upregulation of CYP24A1 expression and, consequently, enhanced catabolism of endogenous 1,25(OH)_2_D_3_. The diminished signaling from endogenous 1,25(OH)_2_D_3_-bound VDR is likely compensated for—and potentially even amplified—by the VDR bound to the synthetic analog. Notably, serum levels of 25-hydroxyvitamin D_3_ remain unaffected under such conditions. Importantly, no severe adverse effects were reported during three years of continuous eldecalcitol administration in clinical settings. Therefore, it is conceivable that **PRI-1938** may similarly lower serum 1,25(OH)_2_D_3_ levels in clinical applications, but the risk of this leading to serious side effects appears to be low.

MD simulations combined with MM-PBSA calculations corroborated the metabolic data, establishing a clear correlation between binding free energy and metabolic resistance. **PRI-1938** consistently exhibited the strongest binding affinities toward both *h*CYP3A4 and *r*CYP24A1, whereas **PRI-1927** showed the weakest interactions. Structure–activity relationship (SAR) analysis pinpointed critical structural determinants governing these properties: A-ring substituent patterns, side chain modifications (notably the 19-methylene at C4 and 29-methyl), and stereochemical configuration at C22 and C24. Detailed analysis of ligand–enzyme interactions within *r*CYP24A1 revealed that **PRI-1938** forms an exceptionally stable binding conformation, stabilized by four key hydrogen bonds involving hydroxyl groups at C1, C3, and C25, and extensive hydrophobic contacts mediated by its side chain methyl and CD-ring system. This optimized interaction network effectively shields the catalytic heme group, resulting in the lowest metabolic conversion rate (5.2%) within the series. By comparison, the less extensive and less spatially coherent interactions formed by **PRI-1927** and **PRI-1937** correlate with their reduced binding free energies and metabolic stabilities.

Overall, these findings demonstrate how precise modifications to both the triene system, and the side chain can dramatically enhance metabolic resistance and binding affinity of vitamin D_3_ analogs. Despite the distinct active site architectures of *h*CYP3A4 and *r*CYP24A1, both enzymes display similar SAR patterns, underscoring the importance of conserved molecular determinants in modulating ligand–enzyme interactions. These insights provide a valuable tip for the rational design of metabolically stable and therapeutically promising vitamin D_3_ derivatives. Further studies on the functional activity of the new analogs—including their affinity for the VDR using a fluorescence-based assay, calcium activity in vitro and in vivo, as well as anticancer activity in leukemia and ovarian cancer cell lines in vitro, and prostate cancer models both in vitro and in vivo—are currently underway in collaborating laboratories.

## Figures and Tables

**Table 1:** The experimental metabolic conversion of 1,25D3 and its analogs by CYP3A4 and CYP24A1 and the calculated free energy of binding to both cytochromes.

Vitamin D Analogs	CYP24A1		CYP3A4	
	Metabolic Conversion by *h*CYP24A1 [% ± SD]	*r*CYP24A1 ΔG*_bind_* [kcal/mol]	Metabolic Conversion by *h*CYP3A4 [% ± SD]	*h*CYP3A4 ΔG*_bind_*[kcal/mol]
**PRI-1927**	14.2 ± 0.8	−56.97	16.7 ± 1.3	−51.80
**PRI-1937**	11.9 ± 1.1	−74.37	16.3 ± 3.7	−70.61
**PRI-1938**	5.2 ± 1.3	−124.54	1.6 ± 1.2	−86.06
**PRI-1906**	4.4 ± 0.7	−93.49	6.2 ± 2.1	−75.80
**1,25D3**	40.0 ± 0.8	−12.91	14.9 ± 3.8	−28.93

## Data Availability

Data is contained within the article.

## Supplementary Materials

The following supporting information can be downloaded at https://www.mdpi.com/article/10.3390/biom15091222/s1. Figure S1. UV Spectrum of PRI-1938 (CH_3_CN:H_2_O 1:1): λ_max_= 241.8 and 273.6 nm; Figure S2. UV Spectrum of PRI-1937 (CH_3_CN:H_2_O 1:1): λ_max_= 274.8 nm; Figure S3. UV Spectrum of PRI-1927 (CH_3_CN:H_2_O 1:1): λ_max_= 266.5 nm; Figure S4. ^1^H NMR of PRI-1927 (500 MHz, CD_3_OD), chemical shifts for ^1^H NMR are given relative to the TMS signal at δ = 0.0 ppm; Figure S5. ^13^C NMR of PRI-1927 (125 MHz, CD_3_OD), chemical shifts for ^13^C NMR are given relative to the TMS signal at δ = 0.0 ppm; Figure S6. ^1^H NMR of PRI-1937 (500 MHz, CD_3_OD), chemical shifts for ^1^H NMR are given relative to the TMS signal at δ = 0.0 ppm; Figure S7. ^13^C NMR of PRI-1937 (125 MHz, CD_3_OD), chemical shifts for ^13^C NMR are given relative to the TMS signal at δ = 0.0 ppm; Figure S8. ^1^H NMR of PRI-1938 (500 MHz, CD_3_OD), chemical shifts for ^1^H NMR are given relative to the TMS signal at δ = 0.0 ppm; Figure S9. ^13^C NMR of PRI-1938 (125 MHz, CD_3_OD), chemical shifts are given relative to the TMS signal at δ = 0.0 ppm.; Figure S10. HPLC profiles of 1,25D3 and its analogs PRI-1937 and PRI-1927 and their metabolites generated by *h*CYP3A4. The peaks marked with arrows indicate putative metabolites; Figure S11. HPLC profiles of analogs PRI-1927, PRI-1938, and PRI-1906 and their metabolites generated by *h*CYP24A1. The peaks marked with arrows indicate putative metabolites. Figure S12. Close-up view of the active site of the rat CYP24A1 structure after molecular docking. Binding of 1,25D3 analogs (PRI-1906, PRI-1927, PRI-1937, and PRI-1938) in the active site of *r*CYP24A1. PRI-1906 (C atoms shown as orange), PRI-1927 (C atoms shown as green), PRI-1937 (C atoms shown as pink), and PRI-1938 (C atoms shown as blue). Surface hydrophobicity is depicted by shaded colors: negative values (blue) correspond to hydrophilic residues, while positive values (brown) correspond to hydrophobic residues. Figure S13. Close-up view of the active site of the human CYP3A4 structure after molecular docking. Binding of 1,25D3 analogs (PRI-1906, PRI-1927, PRI-1937, and PRI-1938) in the active site of *h*CYP3A4. PRI-1906 (C atoms shown as orange), PRI-1927 (C atoms shown as green), PRI-1937 (C atoms shown as pink), and PRI-1938 (C atoms shown as blue). Surface hydrophobicity is depicted by shaded colors: negative values (blue) correspond to hydrophilic residues, while positive values (brown) correspond to hydrophobic residues.
