# Supplementary material for: Metabolic Interactions of Side-chain Extended and Unsaturated Vitamin D Analogs with Cytochrome P450 Enzymes: Integrating Theoretical and Experimental Approaches
Source: Biomolecules. 2025 Aug 25;15(9):1222. doi: 10.3390/biom15091222 (PMC12467546; doi:10.3390/biom15091222)
Supplement: Supplementary file 1 [file biomolecules-15-01222-s001.zip › biomolecules-3805169-supplementary.pdf]

Supplementary material

Metabolic Interactions of Side-chain Extended and Unsaturated Vitamin D Analogs with Cytochrome P450 Enzymes: Integrating Theoretical and Experimental Approaches

Teresa Żołek[a,§,*], Mayur Kadam[b,§], Sharmin Nadkarni[c], Kaori Yasuda[d,§], Michał Chodyński[e], Krzysztof Krajewski[e], Olga Michalak[e], Joanna Tobiasz[e], Marek Kubiszewski[e], Toshiyuki Sakaki[d,‡] and Andrzej Kutner[b,‡,*]

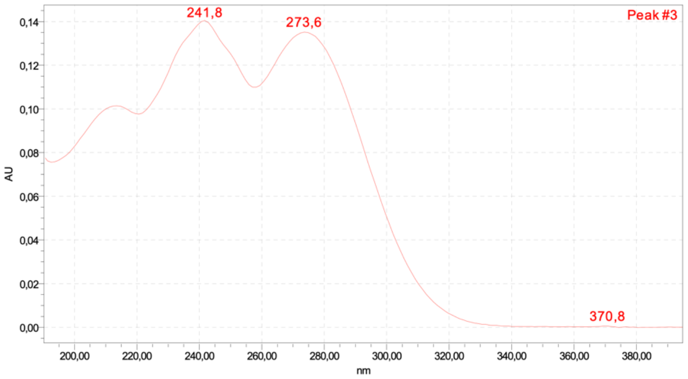

Figure S1. UV Spectrum of **PRI-1938** (CH_3CN:H_2O 1:1): λ_{max}= 241.8 and 273.6 nm.

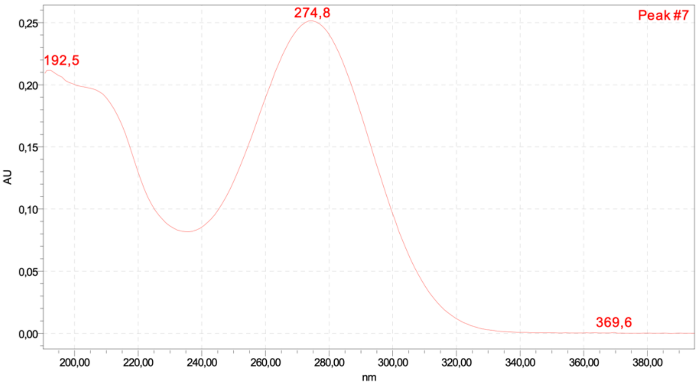

Figure S2. UV Spectrum of **PRI-1937** (CH_3CN:H_2O 1:1): λ_{max}= 274.8 nm.

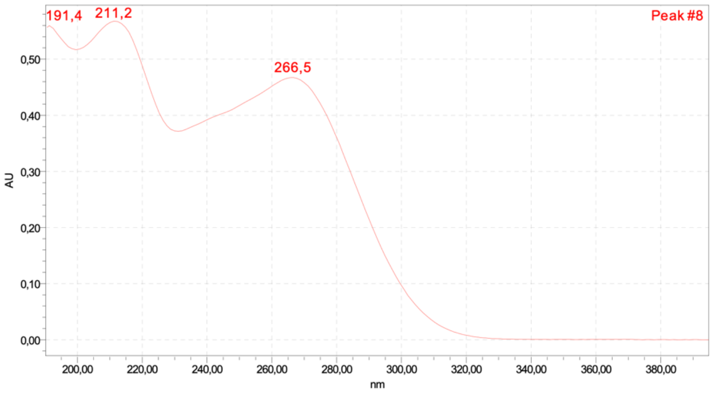

Figure S3. UV Spectrum of **PRI-1927** (CH_3CN:H_2O 1:1): λ_{max}= 266.5 nm.

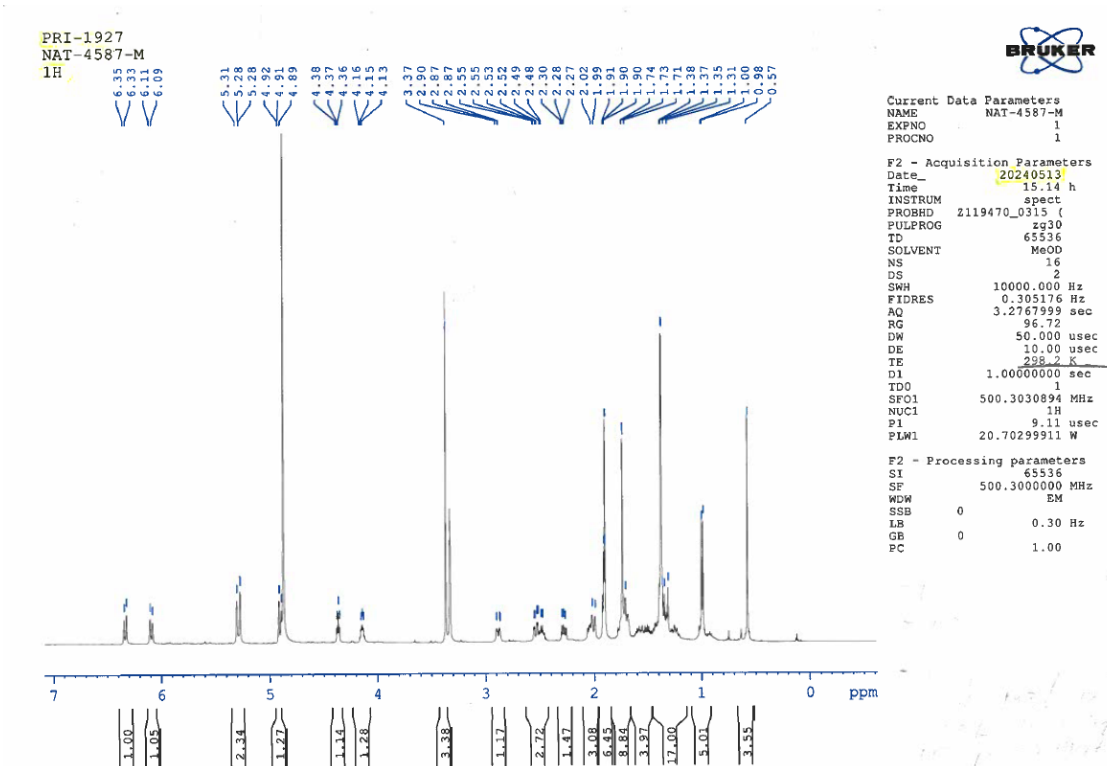

Figure S4. **^{1}H NMR** of **PRI-1927** (500 MHz, CD_3OD), chemical shifts for **^{1}H NMR** are given relative to the TMS signal at δ = 0.0 ppm.

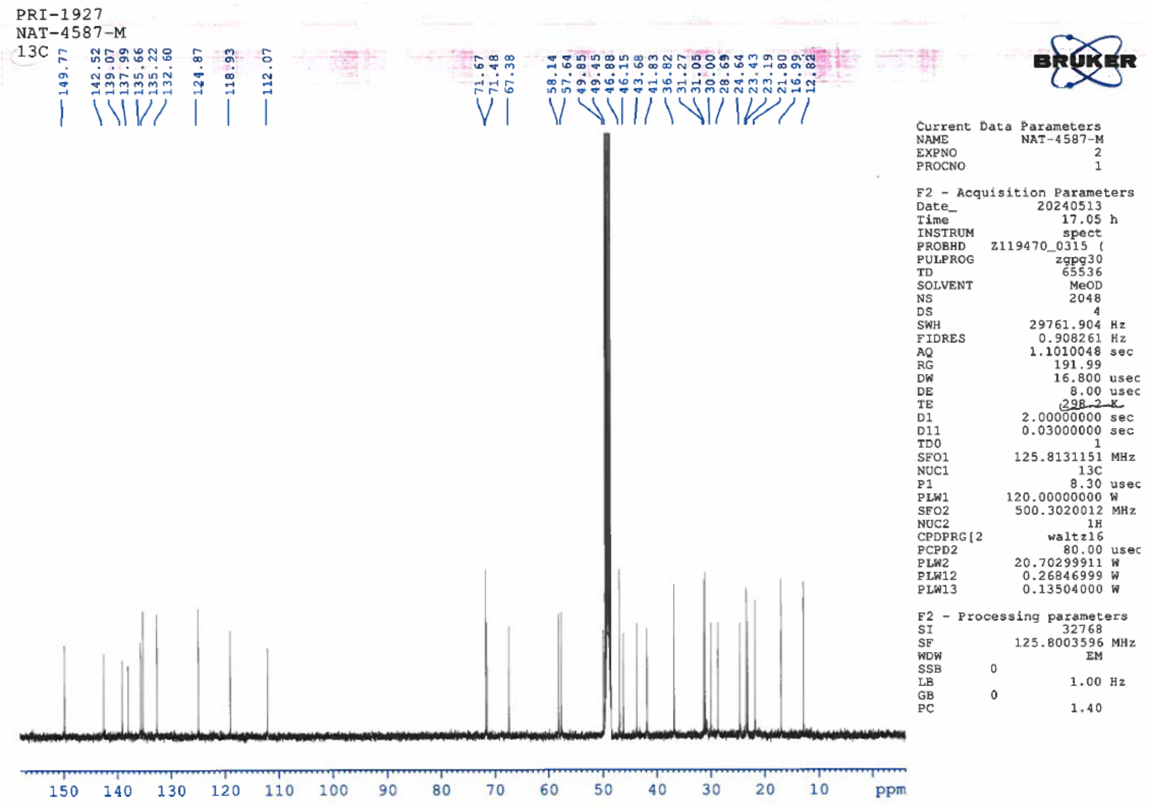

Figure S5. **^{13}C NMR** of **PRI-1927** (125 MHz, CD_3OD), chemical shifts for **^{13}C NMR** are given relative to the TMS signal at δ = 0.0 ppm.

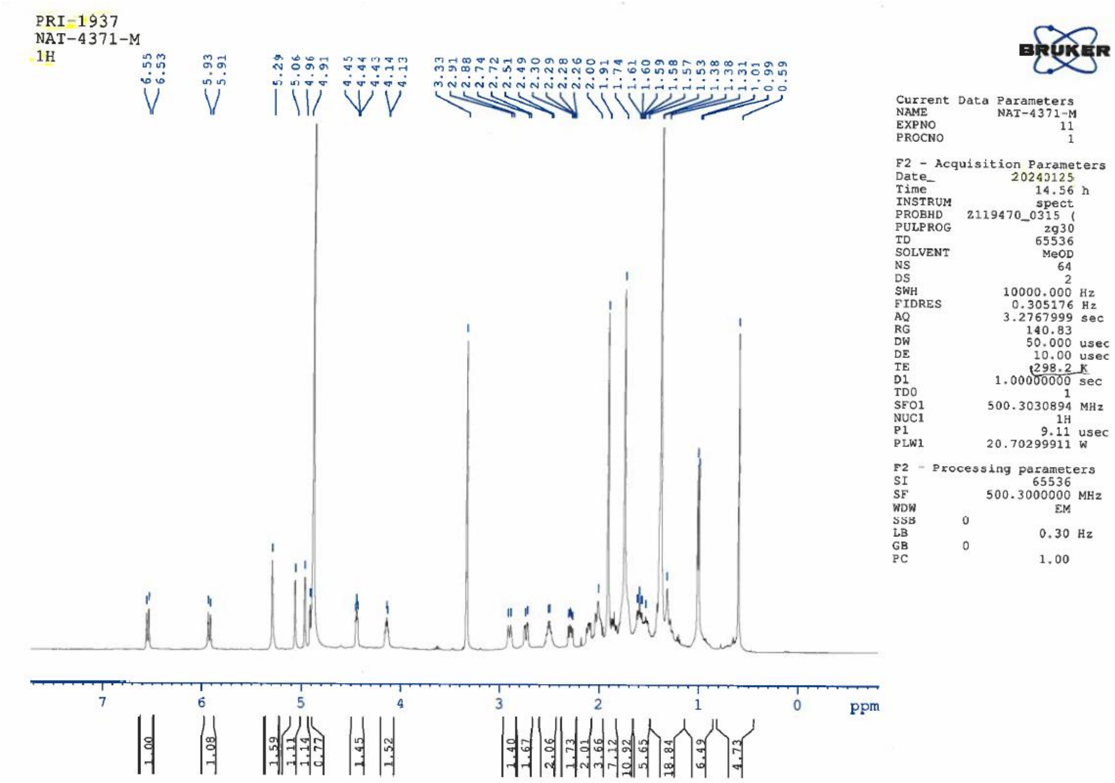

Figure S6. **^{1}H NMR** of **PRI-1937** (500 MHz, CD_3OD), chemical shifts for **^{1}H NMR** are given relative to the TMS signal at δ = 0.0 ppm.

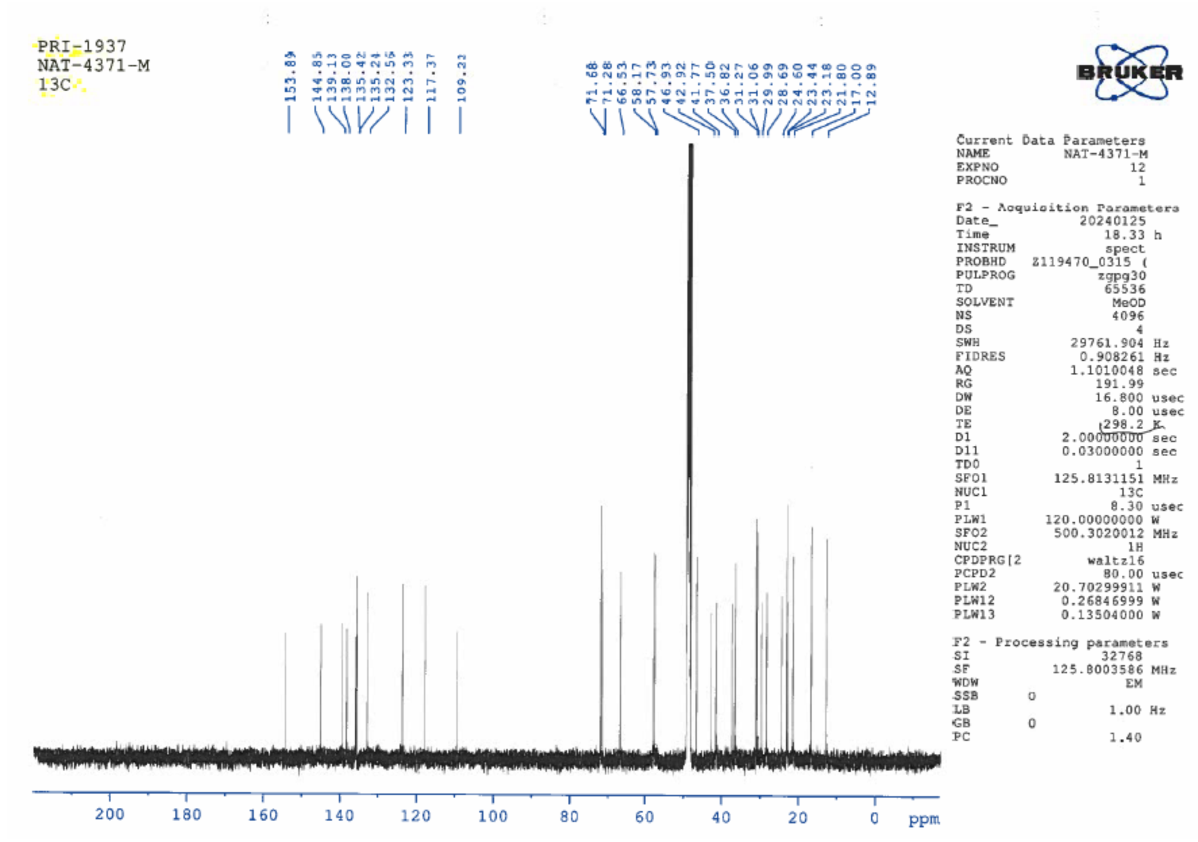

Figure S7. **^{13}C NMR** of **PRI-1937** (125 MHz, CD_3OD), chemical shifts for **^{13}C NMR** are given relative to the TMS signal at δ = 0.0 ppm.

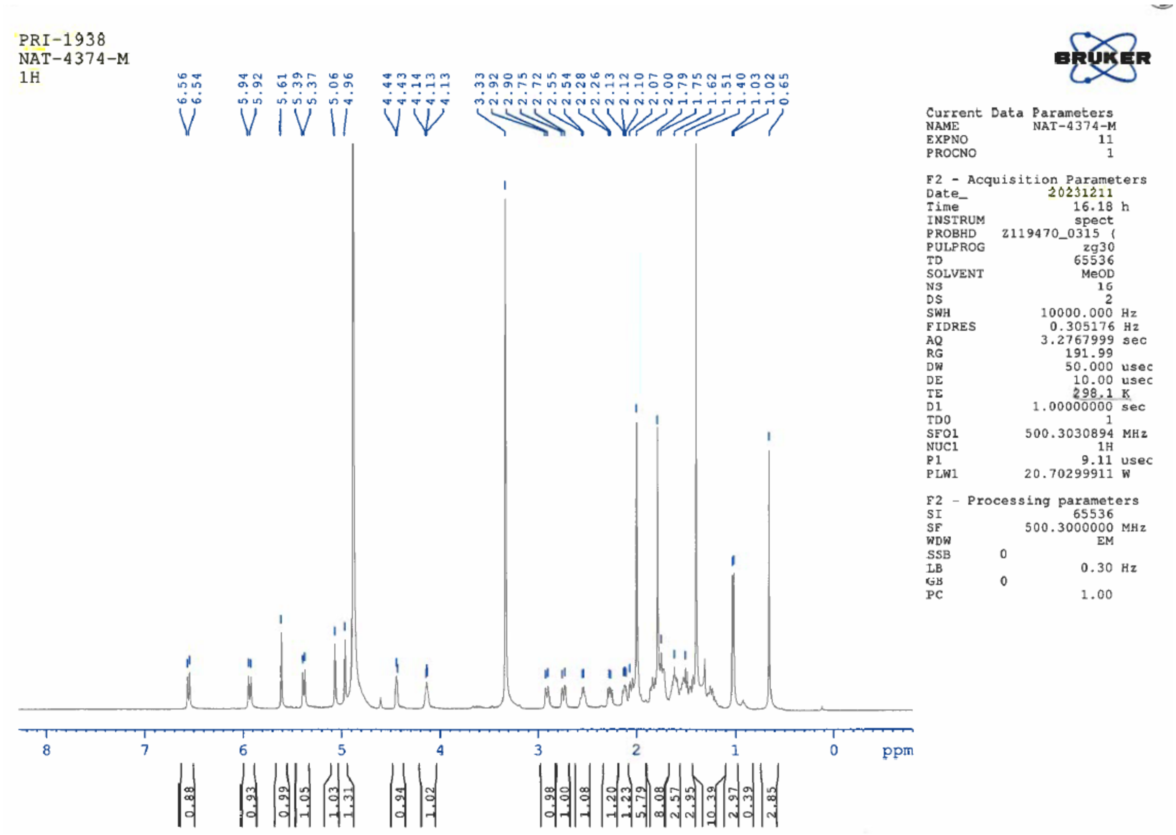

Figure S8. ^{1}**H NMR** of **PRI-1938** (500 MHz, CD_3OD), chemical shifts for ^{1}**H NMR** are given relative to the TMS signal at δ = 0.0 ppm.

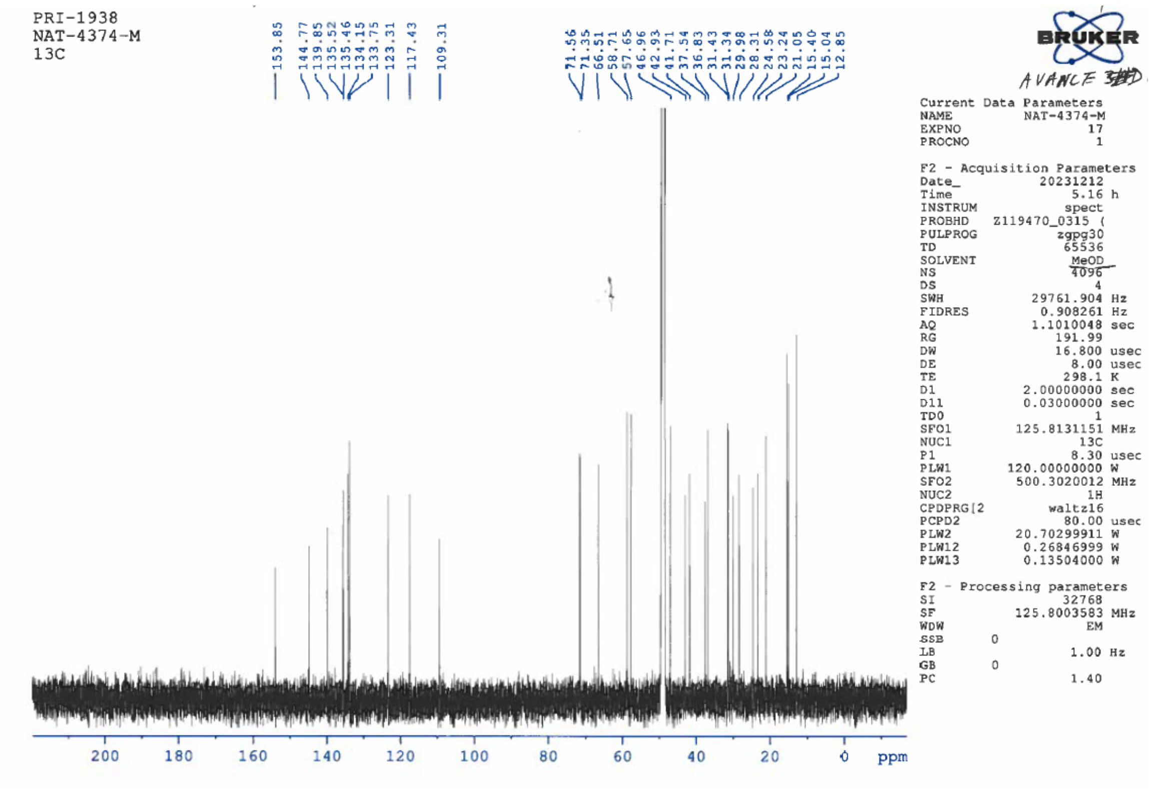

Figure S9. ^{13}**C NMR** of **PRI-1938** (125 MHz, CD_3OD), chemical shifts are given relative to the TMS signal at δ = 0.0 ppm.

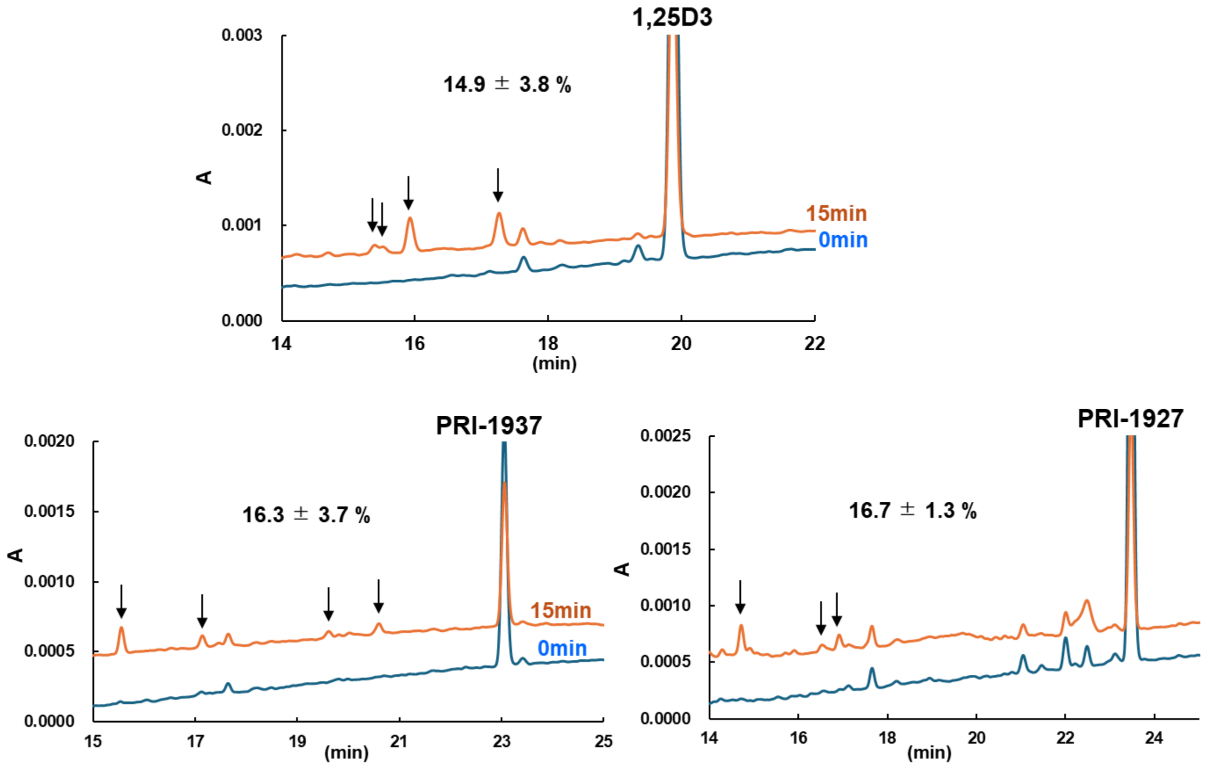

Figure S10. HPLC profiles of **1,25D3** and its analogs **PRI-1937** and **PRI-1927** and their metabolites generated by *h*CYP3A4. The peaks marked with arrows indicate putative metabolites.

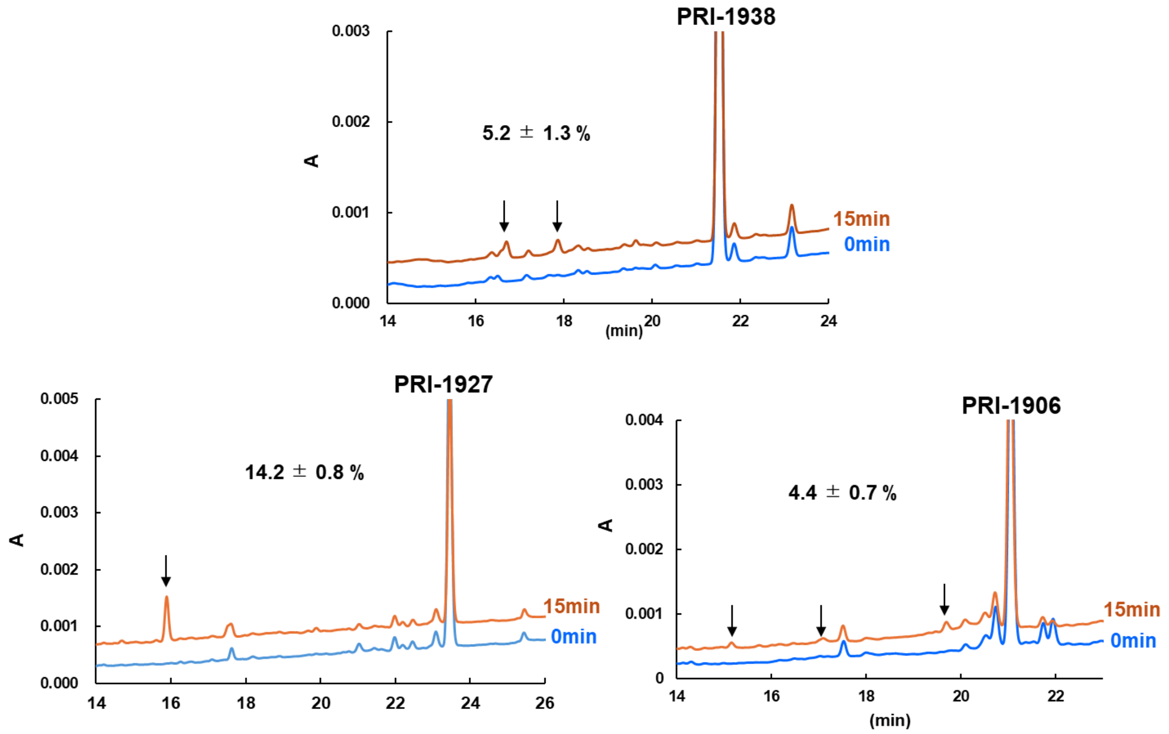

Figure S11. HPLC profiles of analogs **PRI-1927, PRI-1938,** and **PRI-1906** and their metabolites generated by *h*CYP24A1. The peaks marked with arrows indicate putative metabolites.

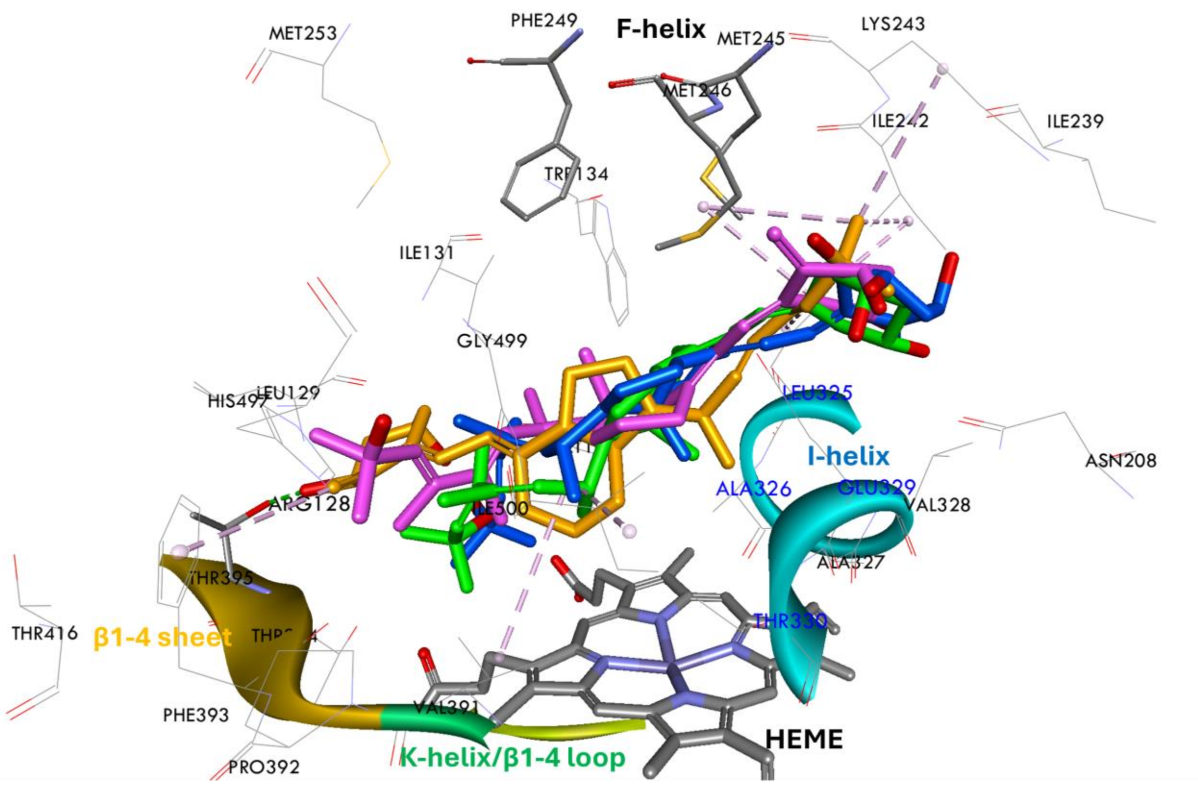

Figure S12. Close-up view of the active site of the rat CYP24A1 structure after molecular docking. Binding of 1,25D3 analogs (**PRI-1906**, **PRI-1927**, **PRI-1937**, **and PRI-1938**) in the active site of *r*CYP24A1. **PRI-1906** (C atoms shown as orange), **PRI-1927** (C atoms shown as green), **PRI-1937** (C atoms shown as pink) and **PRI-1938** (C atoms shown as blue). Surface hydrophobicity is depicted by shaded colors: negative values (blue) correspond to hydrophilic residues, while positive values (brown) correspond to hydrophobic residues.

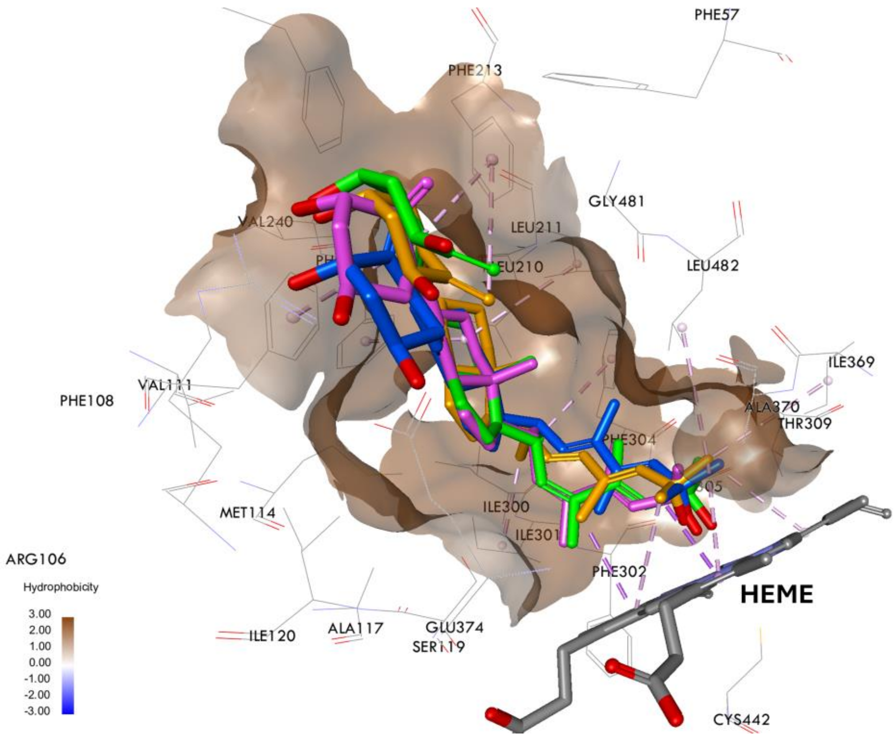

Figure S13. Close-up view of the active site of the human CYP3A4 structure after molecular docking. Binding of 1,25D3 analogs (**PRI-1906**, **PRI-1927**, **PRI-1937,** and **PRI-1938**) in the active site of *h*CYP3A4. **PRI-1906** (C atoms shown as orange), **PRI-1927** (C atoms shown as green), **PRI-1937** (C atoms shown as pink), and **PRI-1938** (C atoms shown as blue). Surface hydrophobicity is depicted by shaded colors: negative values (blue) correspond to hydrophilic residues, while positive values (brown) correspond to hydrophobic residues.
